# Heavy Metal Chelation and Bioprotective Activity of Dextran-DOTA Macromolecular Conjugate

**DOI:** 10.1021/acs.biomac.6c00847

**Published:** 2026-08-05

**Authors:** Sebastian Guajardo, Hunter Wood, Chenyun Deng, Neo Alpha, Stefanie A. Sydlik

**Affiliations:** † Department of Chemistry, Carnegie Mellon University, Pittsburgh, Pennsylvania 15213-2617, United States; ‡ Department of Biomedical Engineering, Carnegie Mellon University, Pittsburgh, Pennsylvania 15213-3890, United States

## Abstract

Widespread exposure and bioaccumulation of toxic heavy metals have increased the incidence of metal poisoning, particularly in children. Although chelation therapy can remove toxic metals, current drugs are limited by toxicity and side effects, restricting treatment to severe cases. We report the synthesis of a water-soluble macromolecular chelator through esterification of dextran with tetraxetan (DOTA) to create a safer systemic chelator. Chemical characterization confirmed successful conjugation with a high degree of substitution. The resulting Dex-DOTA conjugate exhibited enhanced affinity for lead and cadmium, demonstrating the advantages of macromolecular architecture for metal selectivity and retention. Dex-DOTA also showed excellent colloidal stability, no cytotoxicity, and improved cell survival following metal exposure. In C. elegans, the conjugate was nontoxic and mitigated heavy metal-induced toxicity. These findings establish Dex-DOTA as a stable, selective, and biocompatible chelator with strong potential for safer systemic treatment of heavy metal poisoning.

## Graphical abstract

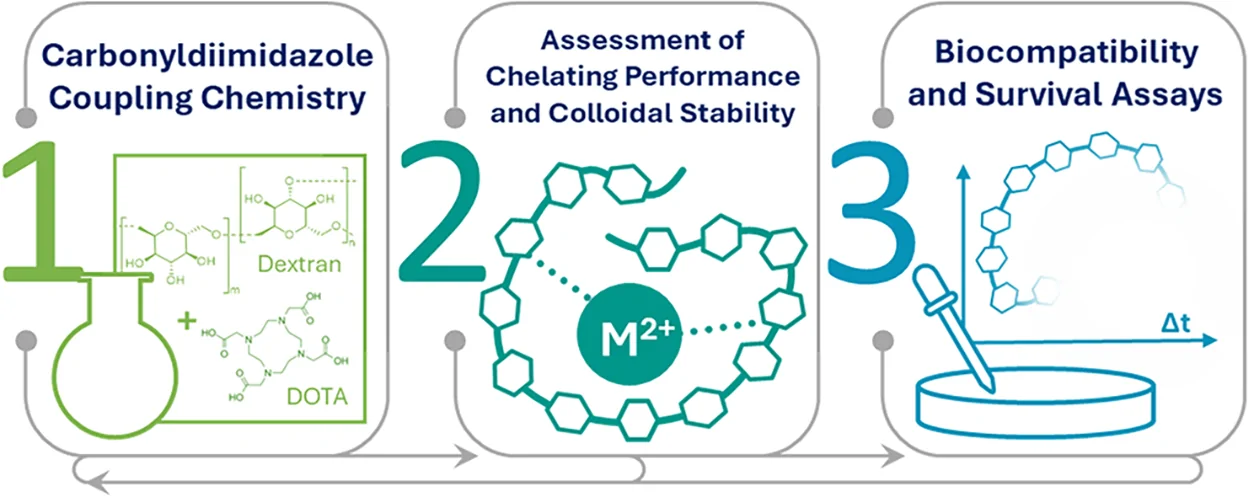

## 1 Introduction

In recent years, heavy metal pollution has emerged as a critical threat to global public health. The World Health Organization (WHO) has identified metal toxins, such as cadmium and lead, among the ten chemicals of major public health concern,^1,2^ with special vulnerability in pediatric populations.^3^ Following ingestion or inhalation, these nonessential metals bioaccumulate within sensitive organs, leading to severe physiological and neurological effects, including renal dysfunction, hepatic failure, reproductive disorders, and neurodegenerative diseases, among other health conditions.^4,5^

Blood concentrations of toxic heavy metals are widely used to assess exposure. In this context, the U.S. Centers for Disease Control and Prevention (CDC) indicates that no blood concentration has been identified below which adverse health effects do not occur. For instance, lead concentrations below 5 μg/dL (50 ppb) have been clinically correlated with diminished cognitive performance, attention deficits, and behavioral disorders in pediatric populations.^6,7^

For several decades, small molecule chelating agents have served as the clinical standard for systemic detoxification in cases of heavy metal poisoning. These small molecules operate by forming stable coordination complexes of metal cations with proximal electron-donor atoms like sulfur, nitrogen, and oxygen. This further facilitates the metabolic elimination of these metal toxins.^8,9^ One of these small molecules is 2,3-dimercapto-1-propanol, also known as British anti-Lewisite (BAL), first used as an antidote to the arsenic-containing warfare agent Lewisite and subsequently used for the treatment of gold and mercury poisoning. Following the development of BAL, other small molecules such as meso-2,3-dimercaptosuccinic acid (DMSA or succimer), sodium 2,3-dimercaptopropane 1-sulfonate (DMPS), and d-penicillamine (DPA) were introduced for clinical use.^9^

While traditional chelation therapy has been effective at reducing the immediate systemic burden of heavy metals, conventional small-molecule therapies are unsuitable for the management of low-to-moderate chronic exposure. Prolonged or high-dose chelation treatments are limited by severe adverse side effects, unfavorable pharmacokinetics, and reduced efficacy.^10,11^ Furthermore, clinical outcomes are often complicated by rapid rebounds upon treatment cessation, caused by the mobilization of metal toxins from the skeletal system or other soft tissues.^12^ Consequently, these limitations create the need for a safer chelator suitable for early intervention and long-term preventive chelation therapies.

Macrocyclic chelators such as 1,4,7,10-tetraazacyclododecane-1,4,7,10-tetraacetic acid (DOTA) present a robust alternative for metal sequestration. This macrocyclic chelator is predominantly recognized as a key component of Lutetium Lu_177_ Dotatate, the first FDA approved radiopharmaceutical therapy for pediatric patients, and for its utility for molecular bioimaging as a contrast enhancement agent.^13–15^ Notably, beyond its high stability with trivalent lanthanide ions, DOTA exhibits strong binding affinity for divalent heavy metal cations, including lead and cadmium.^16,17^ This capability to form stable water-soluble complexes positions DOTA-derived systems as promising candidates for the selective scavenging of toxic heavy metals from biological systems.

To modulate the in vivo pharmacokinetics and optimize renal clearance rates of metal-DOTA imaging complexes, this chelator has been successfully conjugated to a variety of peptide sequences.^14,18^ Nonetheless, natural polysaccharides have been used as an alternative to peptides in the development of bioconjugated systems, owing to their inherent biocompatibility and biodegradability. Among these sugars, dextran, a neutral extracellular bacterial polysaccharide composed of d-glucopyranose units, has been widely explored in biomedical applications because of its nonimmunogenicity and nonantigenicity.^19,20^ The covalent conjugation of therapeutic agents to the dextran backbone have been shown to improve their water solubility, mitigate their systemic side effects, and enhance the pharmacokinetics and biodistribution of the drugs.^21^

Synthetic strategies for these bioconjugates involve multiple coupling methodologies,^14,22^ typically leveraging side-chain nucleophiles on the polymeric backbone or direct activation of the carboxylic acid groups to facilitate stable covalent attachment. Among these methods, carbodiimide-mediated coupling represents a common synthetic strategy for the conjugation of complex carboxylic acids with alcohols or amines under mild conditions.^23^ In a standard coupling reaction, carboxylic moieties are activated by the carbodiimide reagent, typically *N*,*N*′-dicyclohexylcarbodiimide (DCC) or 1-Ethyl-3-(3-(dimethylamino)­propyl)­carbodiimide (EDC), followed by a nucleophilic attack to form the final conjugate, and urea-derivates as byproducts.^24^

Alternatively, *N*,*N*-carbonyldiimidazole (CDI) has emerged as a highly efficient, nontoxic activating reagent for bioconjugation. Compared to traditional carbodiimide-mediated coupling, CDI-activation demonstrates higher relative conjugation efficiency of complex carboxylic acids to several peptides and polysaccharides while avoiding undesired side reactions,^19,25^ including those with sterically hindered nucleophiles such as in dextran.^20,26^ Furthermore, the low toxicity of CDI and its byproducts render this activator as a preferred choice for the synthesis of complex bioderivatives.^27^ Therefore, this chemistry enables the development of nontoxic macromolecular chelators suitable for oral administration.

While macromolecular systems involving dextran and DOTA have been previously investigated, those studies primarily focused on radiotherapeutic applications through the complexation of radioactive metal ions.^28–30^ To our knowledge, this study represents the first report of a direct conjugation between dextran and DOTA optimized specifically for the chelation of heavy metals. Considering this, we explore the synthesis of a biocompatible, water-soluble macromolecular chelator via CDI-mediated conjugation of DOTA and dextran, capable of effectively sequestering metal toxins. The successful development of this macromolecular conjugate offers a step forward in the pursuit of safer, nontoxic, and long-circulating systemic chelation therapies for metal poisoning.

## 2 Experimental Section

### 2.1 Materials

Dextran T40 and 1-hydroxybenzotriazole (HOBt) were obtained from Tokyo Chemical Industry (Philadelphia, PA, USA). 1,4,7,10-tetraazacyclododecane-1,4,7,10-tetraacetic acid (DOTA) was purchased from AABlocks (San Diego, CA, USA). Cadmium nitrate tetrahydrate, copper sulfate pentahydrate, and pancreatin were obtained from Ward’s Science (St. Catharines, ON, Canada). Pepsin was purchased from Chem-Impex (Wood Dale, IL, USA). Cobalt chloride hexahydrate was purchased from VWR Scientific (Radnor, PA, USA). Potassium phosphate monobasic was purchased from Electron Microscopy Sciences (Morgantown, PA, USA). Lead nitrate, 1,1-carbonyldiimidazole (CDI), phosphate-buffered saline (PBS), dimethyl sulfoxide, ethanol, methanol, hydrochloric acid, and sodium hydroxide were obtained from Thermo Fisher Scientific (Waltham, MA, USA). NIH/3T3 fibroblast and RAW 264.7 macrophage cell lines were purchased from American Type Culture Collection (Manassas, VA, USA). Both cell lines were cultured in DMEM supplied with 100 U/mL penicillin/streptomycin, while media were supplemented with 10% calf serum and fetal bovine serum, respectively. Both cell lines were cultured in an incubator at 37 °C and 5% CO_2_. All materials used for cell cultures were purchased from Thermo Fisher Scientific (Waltham, MA, USA) unless otherwise noted. *Caenorhabditis elegans* (N2 strain) and *E. coli* OP50 were obtained from the Caenorhabditis Genetics Center (Minneapolis, MN, USA). Bacteriological agar and LB Miller powder were purchased from VWR Scientific (Radnor, PA, USA). Peptone was purchased from Ward’s Science (St. Catharines, ON, Canada). Sodium chloride was purchased from Morton Salt. Cholesterol, calcium chloride, and potassium hydrogen phosphate were purchased from Beantown Chemical (Hudson, NH, USA). Potassium phosphate monobasic was purchased from Electron Microscopy Sciences (Morgantown, PA, USA). Magnesium sulfate was purchased from Oakwood Chemical (Estill, SC, USA). Deionized water was used for preparation of all buffers and stock solutions.

### 2.2 Synthesis of Dex-DOTA Conjugate

In a typical synthesis, 972.9 mg of 1,1-carbonyldiimidazole (CDI, 6.0 mmol) is dissolved in 50 mL of DMSO at 80 °C. Once dissolved and at the desired temperature, 404.4 mg of 1,4,7,10-tetraazacyclododecane-1,4,7,10-tetraacetic acid (DOTA, 1.0 mmol) is further added for activation and left under constant stirring for 3 h or until the solution turns brown, without any sign of undissolved DOTA. Following the activation step, 81.1 mg of dextran (0.5 mmol; anhydro glucose unit molecular weight: 162.14 mg mmol^–1^) is added along with a catalytic amount of 1-hydroxybenzotriazole (HOBt, 13.5 mg, 0.1 mmol). The reaction proceeds over 18 h at 80 °C under constant stirring with a condenser to avoid loss of volume. Following the reaction, the product is poured into 300 mL of methanol, letting the product precipitate. Once the supernatant is carefully discarded, the collected precipitate undergoes repeated cycles of ethanol washing and centrifugation. After the product is thoroughly washed, the precipitate is dissolved in 30 mL of DI water and dialyzed against pure DI water over 5 days, changing the DI water daily. Finally, the purified product is frozen and lyophilized. The dried samples were prepared for further chemical characterization (NMR, FTIR, XPS, and SEM-EDX).

### 2.3 Chelating Performance Assessment of the Dex-DOTA Conjugate

In order to evaluate the affinity of the Dex-DOTA macromolecular chelator for different metals, a 50 mM metallic aqueous solution of lead, cadmium, cobalt, or copper were prepared. Then, 300 μL of a metallic solution were mixed with 300 μL of diluted Dex-DOTA (DS ∼ 0.66) at different concentrations (5 mM, 2.5 mM, 0.5 mM, 0.25 mM, and 0.05 mM). The final samples were allowed to stand at room temperature overnight. Finally, 300 μL of each sample was transferred to a 96 well plate and analyzed via UV–visible (UV–vis) spectroscopy. Nonexposed metal and Dex-DOTA dilutions were also analyzed as blank samples. In a complementary experiment, to analyze the effect of the conjugation efficiency over the chelation capacity of the Dex-DOTA conjugate, 300 μL of a 50 mM cadmium solution were mixed with 300 μL of Dex-DOTA solutions at different concentrations (5 mM, 2.5 mM, 0.5 mM, 0.25 mM, and 0.05 mM) and degrees of substitution (pure dextran, DS ∼ 0.15, DS ∼ 0.26, and DS ∼ 0.66). The samples were allowed to stand at room temperature overnight and were further analyzed using UV–vis at a wavelength of 310 nm. Nonexposed cadmium solution and the Dex-DOTA dilutions were also analyzed as blank samples. Lastly, to confirm the improvements of the chelation of heavy metals after conjugation, 300 μL of a Dex-DOTA solution (1.25 mM, DS ∼ 0.99), or monomeric DOTA at equimolar concentration, were mixed with 300 μL of diluted cadmium or lead at different concentrations. The samples were allowed to stand at room temperature overnight and the samples were allowed to stand at room temperature overnight and further analyzed using UV–vis at a wavelength of 290 and 310 nm for lead and cadmium, respectively. Nonexposed metal solutions and DI water were also analyzed as blank samples. All the absorption data was acquired in multiple replicates using a Tecan Spark plate reader with SparkControl v2.2 software, with additional preparation of the corresponding calibration curve for each metal studied.

### 2.4 Colloidal Stability Assessment of the Dex-DOTA Conjugate

In order to analyze the colloidal stability of the Dex-DOTA conjugate, samples of 1 mL (0.1 mM, DS ∼ 0.99) were prepared using different simulated body fluids (Phosphate-buffered saline, simulated gastric fluid, and simulated intestinal fluid) and incubated at 37 °C for different time periods (48 h for PBS, and 6 h for SGF or SIF) in order to emulate the residency of the macromolecule in the corresponding portions of the gastrointestinal tract. The hydrodynamic particle size of the Dex-DOTA and its distribution was obtained in multiple replicates via Dynamic Light Scattering. Preparation of the simulated body fluids: PBS was acquired and used according to the specifications of the vendor. To prepare simulated gastric fluid (SGF), 2.0 g of sodium chloride and 3.2 g of pepsin were dissolved in 7.0 mL of hydrochloric acid. The final solution was diluted to 1000 mL and the pH was adjusted to 1.2 using HCl or NaOH. Similarly, to prepare simulated intestinal fluid (SIF), 6.8 g of monobasic potassium phosphate was dissolved in 250 mL of DI water followed by addition of 77 mL of 0.2 M NaOH and additional 500 mL of DI water. Finally, 10.0 g of pancreatin were added, and diluted to 1000 mL adjusting the pH to 6.8 using diluted NaOH or HCl.

### 2.5 Cytocompatibility of Dex-DOTA Conjugate

Cytocompatibility of the materials was assessed by exposure of two mammalian cell lines to dispersions of different concentrations of the powder. Stock solutions of DOTA and Dex-DOTA were prepared by dissolving materials in PBS at 20× of final experimental concentrations (1 mg/mL, 0.1 mg/mL, and 0.01 mg/mL; 1 mg/mL ∼ 1.8 mM). 190 μL of NIH/3T3 fibroblasts and RAW 264.7 macrophages suspensions were seeded in the interior wells of 96 well plates at densities of 3 × 10^4^ and 2 × 10^4^ cells/cm^2^, respectively. After 2 h, the cells were considered well-adhered. Then, 10 μL of DOTA or Dex-DOTA solution were added to wells in triplicate. A no treatment condition of 10 μL PBS was included for comparison. Cells were allowed to grow in the incubator for 48 h, and then the vitality assays were performed. Cellular enumeration, vitality, and late necrosis and apoptosis were assessed using fluorescent reporters. The media and materials were aspirated from the wells, and the following fluorescent staining solution was added: PBS with 20 μM of Hoechst 33342, 25 μg/mL of Calcein AM, and 10 μg/mL of propidium iodide (Alfa Aesar). After 10 min in the incubator, the staining solution was aspirated then washed with PBS. Cells were then imaged under a fluorescence microscope (EVOS FL Auto, Life Technologies). Images were processed in ImageJ to remove background. Relative live cell counts were calculated in ImageJ, by the strength of green-fluorescent signal in images after background was removed.

### 2.6 In Vitro Survival Studies

Stock solutions of Pb and Cd in 20× of final concentration (0.25 mg/mL) were prepared in sterile 0.9% NaCl saline. Cells were seeded in the same density as mentioned in cytocompatibility procedures, but in 180 μL. After 2 h, the cells were considered well-adhered. Then, 10 μL of DOTA or Dex-DOTA solution at a concentration of 0.5 mg/mL and 10 μL of heavy metal solution were added to wells in triplicate. 10 μL PBS and/or 10 μL of 0.9% NaCl saline were used as no treatment control for DOTA/Dex-DOTA and Pb or Cd, respectively. Cells were cultured and analyzed in the same method as mentioned above.

### 2.7 In Vivo Studies Using *Caenorhabditis elegans* (*C. elegans*) Models

The N2 strain of *C. elegans* and OP50 *E. coli* were obtained from the University of Minnesota Caenorhabditis Genetics Center and maintained at 20 °C according to standard literature protocol.^31^ Nematode growth media agar (NGM) was prepared by combining NaCl (3 g), bacteriological agar (17 g), and peptone (2.5 g) in a 2 L glass bottle, followed by addition of deionized water (975 mL). The flask was loosely capped, covered with aluminum foil, and autoclaved for 1 h. After autoclaving, the medium was cooled to 60 °C in a water bath for 15–20 min before supplementation with CaCl2 (1 mL, 1 M), cholesterol (1 mL, 5 mg/mL in ethanol), MgSO4 (1 mL, 1 M), and potassium phosphate buffer buffer (25 mL, 1 M). The medium was gently swirled to ensure homogeneity and either used immediately to prepare agar plates or stored at 60 °C for future use. LB Miller broth was prepared by weighing 2.5 g of LB Miller powder into a 150 mL glass bottle followed by 100 mL of deionized water. The bottle was loosely capped, covered with aluminum foil, and autoclaved for 1 h. Following the autoclave, the solution was left to cool to room temperature before use. LB Miller agar was prepared by adding 5 g of LB Miller powder, 3 g of bacteriological agar, and 200 mL of deionized water to a 250 mL glass bottle. The bottle was loosely capped, covered with aluminum foil, and autoclaved for 1 h. Following the autoclave, the medium was left to cool in a water bath for 15–20 min to reach 60 °C, at which point it was either used to prepare agar plates, or stored for future use.

A starter culture of *E. coli* OP50 was streaked onto LB Miller agar plates to isolate single colonies. A single colony was then aseptically transferred into sterile LB Miller broth and incubated overnight at 37 °C with shaking to generate a liquid OP50 stock culture. The resulting culture was used directly for seeding NGM plates. Both OP50 streak plates and liquid cultures were stored at 4 °C and remained usable for several months. Synchronization of *C. elegans*: *C. elegans* cultured on nematode growth media (NGM) were rinsed from 4 mm × 60 mm plates into a sterile 15 mL centrifuge tube using sterile water. The worms were centrifuged at 400*g* for 1 min and the supernatant was aspirated. Next, the worms were rinsed by consecutive redispersal, centrifugation, and aspiration using 5 mL of sterile water. *C. elegans* were then exposed to a 1:2 v/v ratio of 5 M NaOH and bleach to dissolve the worm cuticle which allows the worm eggs to be isolated. Tubes were vortexed every 2 min while the rate of worm dissolution was monitored by a microscope. The cuticle dissolution process was stopped once most worm bodies had dissolved (6–8 min) by quickly filling the centrifuge tube with sterile M9 non-nutrient buffer and centrifuging at 1300*g* for 1 min. The supernatant was aspirated, and the pelleted eggs were rinsed three times by consecutive redispersal, centrifugation, and aspiration using 5 mL of sterile M9 buffer for each rinse. Eggs were then redispersed in 100 μL of sterile M9 buffer and transferred to a 60 mm NGM plate without an OP50 *E. coli* bacterial lawn. Plates were incubated at 20 °C to allow eggs to hatch, yielding synchronized L1 worms.

Acute liquid exposure assays were performed in a phosphate-free medium consisting of 0.85% NaCl prepared in deionized water in order to maintain lead solubility and minimize precipitation artifacts. All exposure reagents were prepared as 10× concentrated stock solutions to ensure consistent and accurate dosing across wells. Lead nitrate was prepared at 7.0 mM in 0.85% NaCl, and Dex-DOTA was prepared at 0.70 mM in the same buffer. Sodium chloride for the osmotic stress positive control was prepared at 2.0 M. For the combined treatment condition, a single 10× stock containing Dex-DOTA at 0.70 mM and lead nitrate at 7.0 mM was prepared to preserve the intended stoichiometric ratio during administration. Exposure experiments were conducted in sterile 24 well plates at a final volume of 500 μL per well. Each well contained 430 μL of 0.85% NaCl buffer, 10 μL of washed OP50 bacterial suspension, and 10 μL of worm suspension. Exposure was initiated by addition of 50 μL of the appropriate 10× stock solution. Five treatment groups were evaluated, including a negative control consisting of buffer and OP50, Dex-DOTA only at 70 μM, an osmotic stress positive control at 200 mM NaCl, lead only at 0.70 mM Pb­(NO_3_)_2_, and a combined lead and Dex-DOTA treatment containing 0.70 mM Pb­(NO_3_)_2_ with 70 μM Dex-DOTA. In the combined treatment condition, the Dex-DOTA to lead ratio was fixed at 1 to 10 on a molar basis. Worm density was standardized at approximately 20 animals per well. Plates were incubated for approximately 24 h at 20 °C with gentle agitation to reduce settling and maintain uniform exposure conditions.

A complete description of *C. elegans* data analysis and experimental instrumentation can be found in Supporting Information.

## 3 Results and Discussion

To evaluate the potential of Dextran-DOTA macromolecular conjugate (Dex-DOTA) as a safe, orally administered chelation therapy, a comprehensive suite of studies was conducted. This includes chemical characterization, assessment of chelation performance in aqueous media, *ex vivo* stability testing in simulated body fluids, and the assessment of *in vitro* cytotoxicity and *in vivo* biocompatibility. The successful synthesis of the macromolecular conjugate via CDI-mediated coupling was confirmed through primary assessment of the covalent ester formation. Following validation of the covalent attachment, the chelation capacity and physiological stability of the macromolecule were evaluated to determine its efficacy under biomimetic conditions. Finally, the biological profile of the Dex-DOTA conjugate was established through *in vitro* cytotoxicity analysis and *in vivo* biocompatibility assessment using *C. elegans* as a model organism.

### 3.1 Chemical Characterization of the Dex-DOTA Macromolecular Conjugate

The synthesis of a potential macromolecular chelator, Dex-DOTA, was achieved through the covalent conjugation of DOTA to the dextran backbone via carbodiimide-mediated esterification. This process involved the initial activation of the carboxylic acid moieties on the macrocyclic DOTA ligand, followed by a nucleophilic attack from the secondary hydroxyl groups of the d-glucopyranose repeating units within the dextran chain. The reaction was facilitated by 1,1-carbonyldiimidazole (CDI) as the coupling reagent, with catalytic amounts of 1-hydroxybenzotriazole (HOBt) employed to promote efficient ester formation. The successful preparation of the Dex-DOTA conjugate and the integrity of the ester bonds were validated through comprehensive characterization (Figure 1).

**Figure 1:**
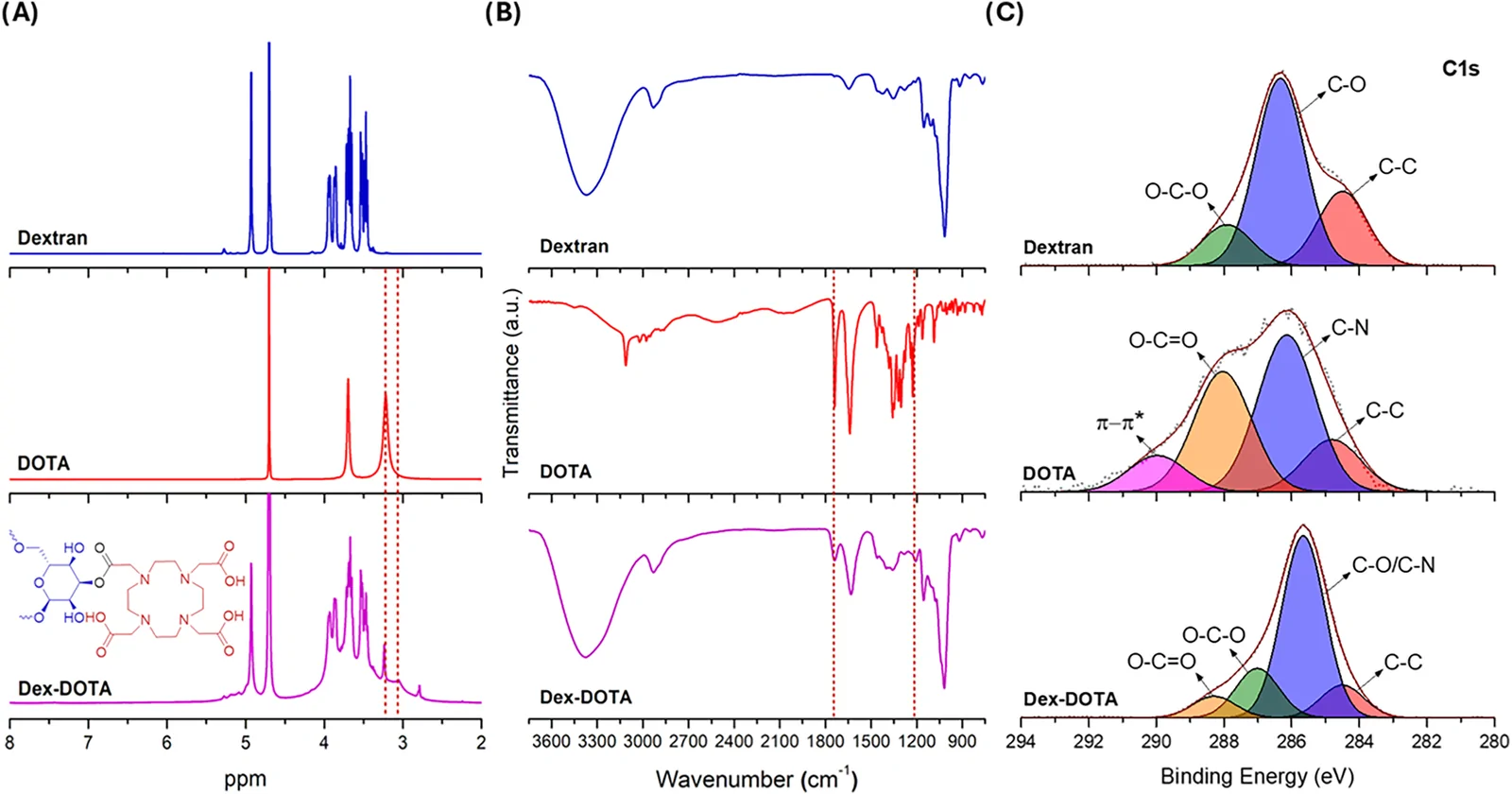
Confirmation of the Dex-DOTA conjugation via chemical characterization. Proton Nuclear Magnetic Resonance (H NMR, A), Fourier Transform Infrared spectroscopy (FTIR, B), and C 1s High-resolution X-ray Photoelectron spectroscopy (XPS, C) for Dextran-DOTA conjugated macromolecule and its precursors, confirming successful CDI coupling reaction of dextran and the chelating molecule.

Validation of the Dex-DOTA covalent conjugation was performed via ^1^H Nuclear Magnetic Resonance spectroscopy (^1^H NMR, Figure 1A). Comparative analysis of the spectra reveals a significant splitting of the resonance peaks associated with the methylene protons of the DOTA cyclen ring. This phenomenon is attributed to the decreased symmetry and altered magnetic environment following covalent attachment. Specifically, a notable upfield shift of these proton signals from 3.22 to 3.06 ppm was observed. This shielding effect is consistent with the modification of the electronic environment induced by the formation of bulky ester groups.^32,33^ These spectral transformations provide evidence for successful conjugation and confirm the partial esterification of the macrocyclic chelator onto the polysaccharide backbone.

Complementary structural evidence was obtained via Fourier-transform Infrared spectroscopy (FTIR, Figure 1B). Comparative analysis of the infrared spectra confirms the successful conjugation of DOTA onto the dextran backbone through the emergence of distinct vibrational modes. Most significantly, a prominent peak appeared at 1740 cm^–1^ corresponding to the C = O stretching vibration of the carbonyl groups from the DOTA carboxylates, indicating the formation of the ester linkage. Additionally, a new vibrational signal at 1210 cm^–1^ was identified which is assigned to the C–N stretching vibration of the aliphatic amines within the DOTA cyclen ring.^26,34^ The presence of these characteristic peaks, absent in the precursor polysaccharide, corroborates the successful covalent attachment of the macrocyclic chelator to the dextran chain.

The formation of the ester bond was further validated through high-resolution X-ray photoelectron spectroscopy (XPS), specifically through deconvolution of the C 1s core-level spectra (Figure 1C). Comparative analysis reveals the emergence of a distinct peak in the Dex-DOTA spectrum assigned to the O–C=O species, a sign of the covalent attachment of the macrocyclic chelator to the polysaccharide backbone. Notably, this O–C=O component exhibited a subtle shift of approximately 0.3 eV in binding energy relative to the carboxylate signal of pristine DOTA. This shift is characteristic of the transition from a free carboxylic acid to an ester, due to the change in surrounding electron density.^35^ Collectively, these spectroscopic data, spanning ^1^H NMR, FTIR, and XPS, provide definitive confirmation of the successful covalent conjugation and chemical integrity of the Dex-DOTA macromolecule.

### 3.2 Conjugation Efficiency for Dex-DOTA Macromolecule

Following validation of the covalent conjugation, the elemental composition and spatial distribution of nitrogen within the conjugate were characterized using Scanning Electron Microscopy coupled with Energy Dispersive X-ray spectroscopy (SEM-EDX, Figure 2). Given the inherent absence of nitrogen in the precursor polysaccharide, the nitrogen signal serves as evidence for the presence of the macrocyclic chelator. By leveraging the relative atomic concentrations of the constituent elements, the degree of substitution (DS) can be estimated, defined as the average number of DOTA molecules per d-glucopyranose repeating unit.

**Figure 2:**
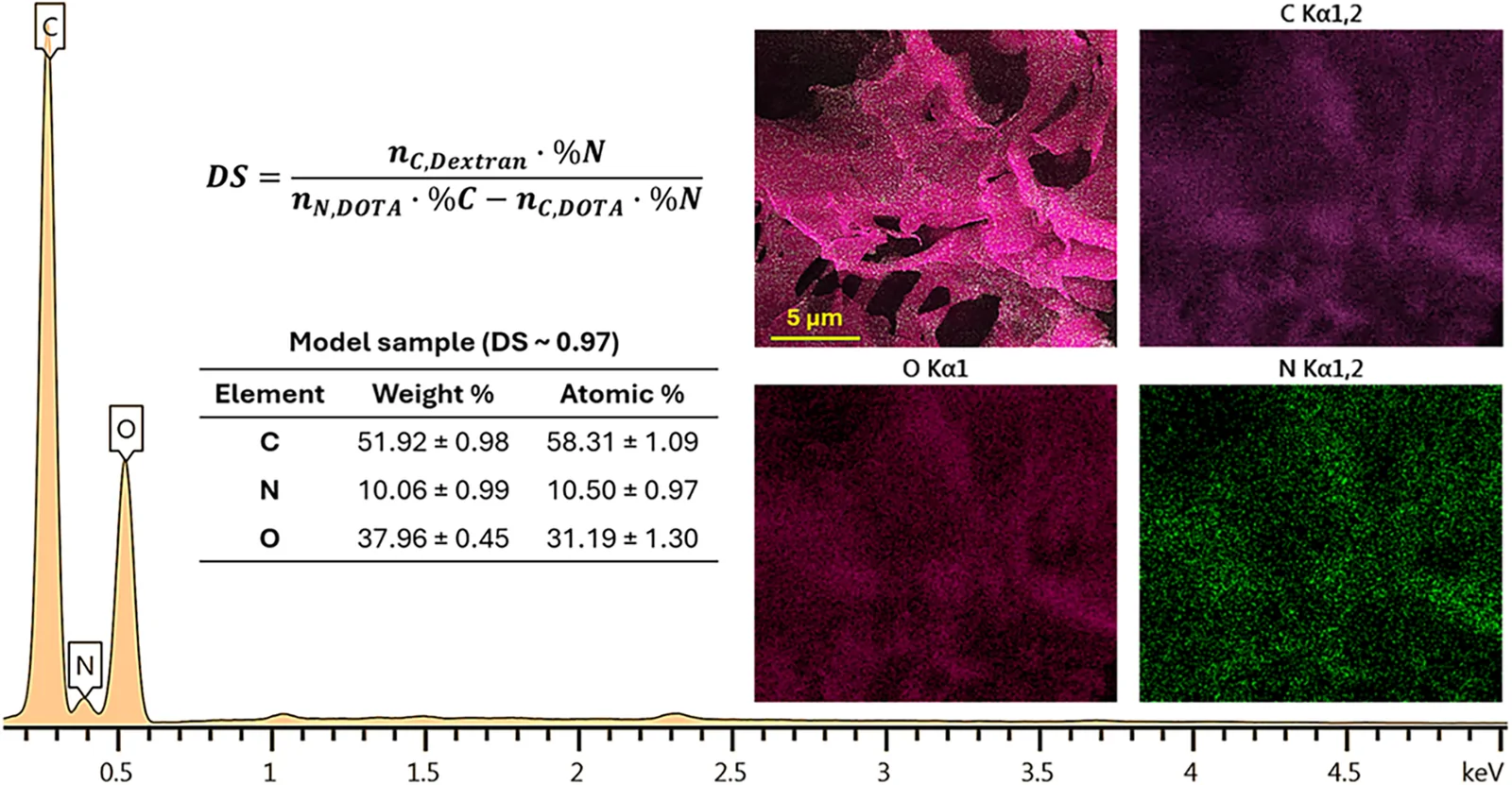
Efficiency of conjugation for Dex-DOTA macromolecule under chelator excess and high activation conditions (molar ratio of chelator, activator, and d-glucopyranose of 1:6:0.5; 3 h of activation at 80 °C). SEM-EDX images for elemental semiquantification and distribution. Degree of substitution is estimated from the embedded formula where n_C,dextran_ represents the number of carbon atoms in a dextran repeated unit, n_C,DOTA_ correspond to the number of carbons in a DOTA molecule, n_N,DOTA_ denotes the number of nitrogen atoms in a DOTA molecule, and %C and %N correspond to the atomic percentage of carbon and nitrogen in the sample, respectively.

Elemental mapping via SEM-EDX (Figure 2) reveals a uniform spatial distribution of nitrogen across the polysaccharide framework for conjugates synthesized under conditions of stoichiometric excess of DOTA and high activation. This homogeneous density of nitrogen suggests random incorporation along the dextran chain. Under these conditions, the degree of substitution was estimated to be 0.99 ± 0.18, meaning approximately one DOTA unit per d-glucopyranose repeated unit. This estimation was based on the atomic concentration of carbon (∼58.31 ± 1.09%) and nitrogen (∼10.50 ± 0.97%) in the sample. These results indicate a high conjugation efficiency, demonstrating the effectiveness of the CDI-mediated activation parameters in achieving dense functionalization of the polysaccharide.

A systemic evaluation varying the synthetic parameters (Table S1) reveals a distinct correlation between reagent stoichiometry and the resulting conjugation efficiency. Specifically, an increase in the molar excess of the macrocyclic chelator and the activating reagent, coupled with prolonged activation of the carboxylic acid moieties, consistently yielded higher degrees of substitution. These observations agree with previous reports, where an excess of chelating molecules and high activation conditions are critical for maximizing the conjugation efficiency in carbodiimide-mediated coupling reactions.^24,36^ Such a trend underscores the controllability of the conjugation process, allowing for precise tuning of the Dex-DOTA macromolecule to meet specific therapeutic requirements.

### 3.3 Assessment of Dex-DOTA Chelating Capacity and Metal Affinity

Upon successful conjugation, Dex-DOTA chelating performance was evaluated through a series of metal sequestration experiments. The concentration of free metal ions in aqueous media was determined via UV–visible spectrophotometry, both prior to and following exposure to the Dex-DOTA macromolecular chelator. To ensure analytical accuracy, external calibration curves were constructed using standard metal salts (Figure S1).

A multiparametric assessment was conducted to elucidate metal ion selectivity (Figure 3A), dependence to conjugation efficiency (Figure 3B), and the impact of the esterification in the chelating performance against heavy metals (Figure 3C). Notably, the Dex-DOTA conjugate shows high solubility in aqueous media. Additionally, no significant physical changes or signs of complex precipitation were observed after heavy metal exposure in any of the evaluated conditions. Representative images of Dex-DOTA dilutions and referential UV–vis spectra of the monomeric and conjugated chelator in aqueous solution are provided in Figure S2.

**Figure 3:**
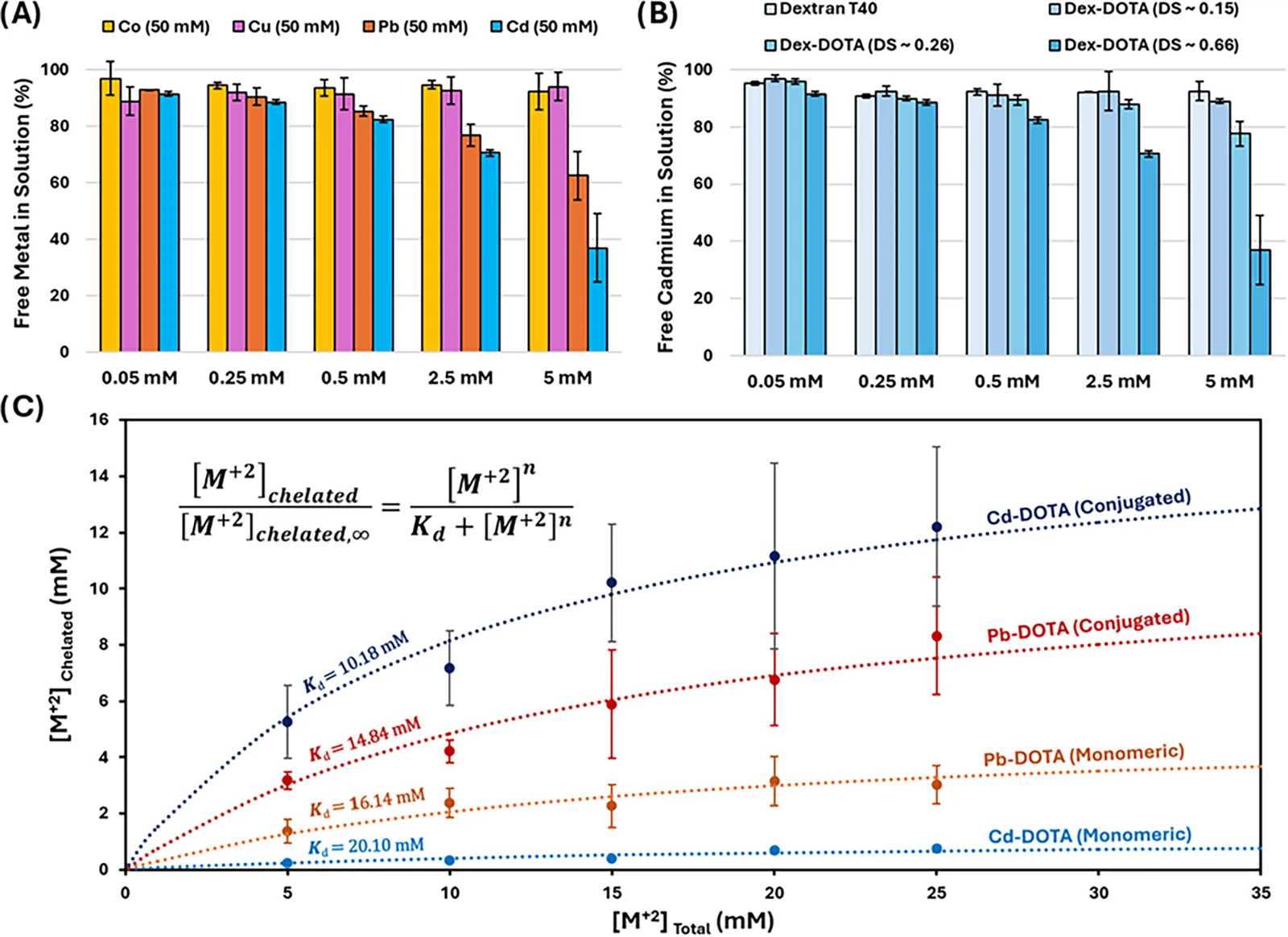
Dex-DOTA chelation activity for common metals involved in poisoning and diseases. Chelating performance of Dex-DOTA (DS ∼ 0.66) via UV–vis analysis at different concentrations over fixed solutions (50 mM) of toxic metals (A), and for different degrees of substitution of Dex-DOTA in a 50 mM cadmium solution (B). Comparison in the chelating performance of DOTA, before and after conjugation, for lead and cadmium (C). Dex-DOTA concentrations are estimated according to the corresponding molecular weight of the substituted anhydrous glycosidic unit based on their degree of substitution. Dex-DOTA (DS ∼ 0.99) was used for direct comparison to monomeric DOTA. The parameters for the binding saturation model were estimated through double-reciprocal analysis followed by nonlinear least-squares regression fitting of the Hill-Langmuir equation.

The metal-binding affinity of the Dex-DOTA conjugate was evaluated through gradual exposure of a range of metals (cobalt, cadmium, lead, and copper) to the macromolecular chelator (Figure 3A). The Dex-DOTA conjugate exhibited a clear concentration-dependent chelating capacity for cadmium and lead, while no significant sequestration was observed for cobalt or copper. Specifically, treatment with 5 mM of Dex-DOTA chelator (DS ∼ 0.66) resulted in a significant reduction of free metal in solution, achieving chelation efficiencies of about 37.5 ± 8.7% and 56.7 ± 10.0% for lead and cadmium, respectively.

The chelation capability of the macromolecular chelator exhibits a strong dependence on concentration and a notable preference for heavier metal toxins, like cadmium and lead, over lighter transition metals, including cobalt and copper. Although DOTA has previously demonstrated good affinity for cobalt^37,38^ and copper,^39^ the modification of the structure via esterification with a polysaccharide can influence the metal coordination affinity. Similar results have been reported, demonstrating that chemical modification of small-molecule chelator structures can lead to significant changes in metal-coordination.^40^

Furthermore, the preferential affinity of the Dex-DOTA to coordinate heavy metals is predominantly governed by the presence of the DOTA macrocycle. This ligand-dependent sequestration mechanism was further tested through subsequent binding assays using cadmium as a representative heavy metal (Figure 3B), which further corroborated the role of the DOTA moiety in facilitating high-affinity coordination for the conjugated macromolecule.

The influence of the conjugation efficiency on metal sequestration was then evaluated by exposing a standardized cadmium solution to Dex-DOTA conjugates with different degrees of substitution (average number of DOTA molecules per d-glucopyranose repeating unit). Macromolecular chelators with a low degree of substitution, below 15 molecules of DOTA per 100 d-glucopyranose repeated units (DS ∼ 0.15), exhibited negligible free metal reduction along the assessed concentration range. Conversely, significant cadmium chelation was achieved as the degree of substitution increased. Specifically, Dex-DOTA macromolecules that possess approximately 66 molecules of DOTA per 100 d-glucopyranose repeated units (DS ∼ 0.66) elicit a reduction of free cadmium in solution of about 56.7 ± 10.0%, confirming that the chelation capacity of the Dex-DOTA is highly dependent on the degree of substitution.

These findings are consistent with the established literature regarding the stability of DOTA complexes, which exhibit higher selectivity for lanthanides and heavy metals over smaller transition metals.^41–43^ Moreover, the hydroxyl groups available for metal coordination from the d-glucopyranose unit exist in equatorial position, which is the less favored arrangement for metal chelation, forming few to no stable complexes.^44,45^ These results corroborate that the macrocyclic DOTA moiety is the primary driver of the chelation process for the Dex-DOTA macromolecule and dictates its preferential affinity for heavy metal toxins.

Furthermore, covalent conjugation of the DOTA macrocycle can significantly influence the overall binding efficacy of the chelator. A comparative analysis of DOTA chelation performance between the free monomeric ligand and the macromolecular conjugate (Figure 3C) indicates that tethering the macrocycle to the dextran backbone significantly enhances its metal-binding capacity. A double-reciprocal analysis followed by nonlinear least-squares regression fitting of the Hill-Langmuir equation was employed to model the binding profiles and determine the maximum chelation capacity and the apparent dissociation constant (*K*_d_) of the conjugates for cadmium and lead (Figure S3 and Table S2). This analysis validates the superior sequestration efficiency of the macromolecular Dex-DOTA conjugate compared to its monomeric counterparts.

Analysis of the saturation curves reveals an improvement in the affinity of the conjugated macromolecule for heavy metal cations compared to its monomeric state. A significant reduction in the apparent dissociation constant, representing the metal concentration at which half-saturation is achieved, underscores the critical role of covalent conjugation in the enhancement of heavy metal chelation. Given that the *K*_d_ value is inversely proportional to the affinity of the chelator for heavy metals, the observed decreases of approximately 8.1% for lead and 49.4% for cadmium relative to monomeric DOTA translate directly into a higher capture efficiency at lower metal concentrations. Consequently, higher saturation values for metal sequestration were also observed, with a 2.1-fold increase for lead and 13.9-fold increase for cadmium after Dex-DOTA conjugation. These results suggest that the chemical coupling of the DOTA macrocycle to the dextran backbone not only increases the binding affinity but also refines the local coordination environment.

This enhanced chelation capacity can be attributed to both local electronic factors and broader structural preorganization. On a molecular level, covalent tethering alters the electronic environment of the ligand while potentially enabling synergistic coordination between the d-glucopyranose units and the DOTA electron donor moieties, thereby stabilizing the complex.^46,47^ Structurally, while monomeric DOTA relies on independent, unconstrained molecular collisions in solution, the Dex-DOTA conjugate arranges these macrocycles along a continuous polymer backbone. This macromolecular network essentially acts as a stabilizing matrix, reducing the systemic fluctuations caused by local, random interactions of free DOTA with heavy metal ions.

Such matrix-stabilized behavior aligns with literature demonstrating that structural modifications and immobilization of macrocyclic chelators can significantly enhance complex stability.^48,49^ Since traditional techniques for mechanistic analysis, such as X-ray crystallography or isothermal titration calorimetry, are not well-suited for polydisperse polymeric substrates, direct elucidation of the complexation mechanism remains challenging. Consequently, further experimental studies are required to understand the underlying synergistic coordination mechanics and isolate the specific electronic contributions of the polymer backbone.

Collectively, these findings demonstrate that integrating high-affinity coordination chemistry with a biocompatible polysaccharide template yields a significant advancement in the design of systemic chelation drugs. While further studies using Inductively Coupled Plasma Mass Spectrometry (ICP-MS) are required to validate the precise sequestration capacity of the Dex-DOTA conjugate within the clinically relevant concentration ranges of blood toxins (*typically*, *parts per billion or μg*/*dL*), this platform offers a highly promising alternative for the safer, more effective management of heavy metal poisoning. This therapeutic potential is further supported by the robust colloidal stability in physiological media, enhanced cytocompatibility, and protective activity observed in our *in vitro* and *in vivo* models.

### 3.4 *Ex Vivo* Stability of Dex-DOTA Macromolecular Chelator under Physiological Conditions

In order to evaluate the colloidal stability of the water-soluble Dex-DOTA conjugate under diverse physiological conditions, the macromolecule was incubated in three different biomimetic media at a controlled temperature of 37 °C. Dynamic light scattering (DLS) was employed to monitor the variations in the hydrodynamic diameter and aggregation of the Dex-DOTA particles following exposure to phosphate-buffered saline (PBS, pH 7.4, Figure 4A), simulated gastric fluid (SGF, pH ∼ 1.2, Figure 4B), and simulated intestinal fluid (SIF, pH ∼ 6.8, Figure 4C). These stability assays were conducted over time intervals corresponding to the respective physiological residence times in the bloodstream and gastrointestinal tract. This systematic characterization ensures the integrity of the macromolecular chelator across different physiological conditions, a prerequisite for effective oral or systemic administration.

**Figure 4:**
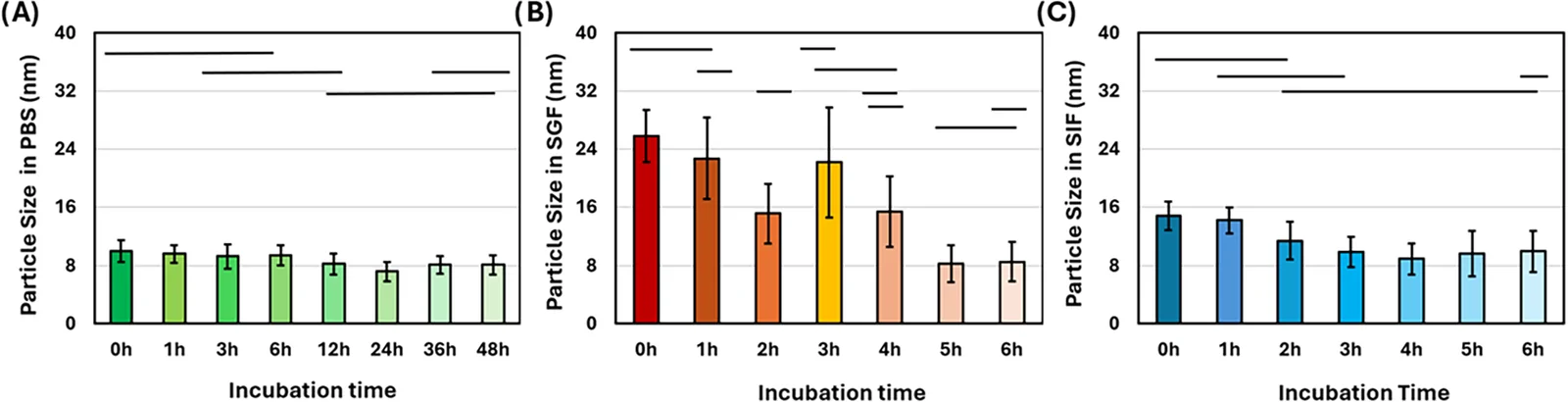
Stability of Dex-DOTA (DS ∼ 0.99) in relevant simulated body fluids. Variation in the particle size after incubation in phosphate-buffered saline (PBS, pH ∼ 7.4, A), simulated gastric fluid (SGF, pH ∼ 1.2, B), and simulated intestinal fluid (SIF, pH ∼ 6.8, C). Upper bars indicate nonsignificant variation among groups (p-value <0.05). The study was carried out over 48 h for PBS to emulate the time of residence in the bloodstream, and 6 h for SGF and SIF to emulate the time of residence in the corresponding portions of the gastrointestinal tract.

Following 48 h of incubation in PBS, the Dex-DOTA conjugate exhibited no significant fluctuations in the hydrodynamic diameter, indicating robust colloidal stability under physiological conditions. This condition serves as a biomimetic model for systemic circulation residence. The observed particle size distribution confirms the consistent formation of discrete nanoconjugates. Notably, the absence of large-scale aggregates throughout the incubation period underscores the high colloidal stability of the macromolecule, suggesting that the Dex-DOTA conjugate remains intact without undergoing undesirable self-association or degradation in the blood-mimicking environment.

Upon incubation in SGF to model gastric residence, a significant increase in the mean hydrodynamic diameter was observed, with average values reaching two to three times those recorded in PBS (Figure 4B). The highly acidic environment characteristic of the gastric lumen (pH ∼ 1.2 to 1.9) induces the formation of higher-order aggregates, which accounted for 10% to 20% of the total volume in the volumetric distribution (Figure S4), within the first hour of exposure.

These species not only shifted the mean particle size but also significantly increased the dispersity of the system. Nevertheless, despite the aggregation of the conjugated macromolecule, the reported value for SGF is the average particle size for over 99% of the particle population by number. Aggregates of ∼1 μm comprise less than 1% of the numerical population; therefore, although their existence cannot be disregarded, the particle size shown in Figure 4B is representative of the majority of the sample upon SGF incubation.

However, continued incubation led to the progressive dissociation of these aggregates, which comprised less than 3% of the total volume after 6 h. This dissolution process resulted in a stabilization of the macromolecular structure after 4 to 5 h, with the final particle size distribution reverting to values comparable to those observed for the conjugate in PBS (8.06 ± 1.35 nm), and unmodified dextran (6.14 ± 0.31 nm, Figure S5).

This behavior is attributed to the drop in pH that neutralizes the negative charge of the carboxylic groups present in the tethered DOTA. Without electrostatic repulsion, hydrophobic interactions dominate, causing the modified polysaccharide to collapse and rapidly clump together into large aggregates. However, the acidic environment induces the breakdown of the macromolecule into smaller fragments over time, causing the large aggregates to physically disintegrate after a few hours.

Similarly, comparable stability was observed during the 6 h incubation of the Dex-DOTA conjugate in SIF (Figure 4C), modeling the transit and residence of the chelator within the small intestine. Initial incubation in SIF resulted in a transient increase in the hydrodynamic diameter compared to PBS values, characterized by the formation of metastable microaggregates. However, these species underwent rapid dissociation under simulated intestinal conditions, with the particle size distribution reverting to values consistent with those observed in PBS within 3 h (9.88 ± 2.07 nm).

Collectively, these results demonstrate that the Dex-DOTA conjugate maintains good colloidal stability across diverse biomimetic environments. This robust stability profile is a critical property for the development of orally administered systemic chelating drugs, ensuring the integrity of the macromolecular conjugate during gastrointestinal and systemic transit.

### 3.5 Cytotoxicity Assessment *In Vitro* for Dex-DOTA Macromolecule

To ensure the viability of the Dex-DOTA conjugate as a safer systemic chelation agent, its biocompatibility is rigorously evaluated. In vitro cytotoxicity assays were conducted using murine fibroblast (NIH/3T3) and macrophage (RAW 264.7) cell lines, which serve as representative models for connective tissue and immune system responses, respectively. The influence of the macromolecular chelator on the cellular homeostasis was quantified by monitoring metabolic proliferation, cell viability, and overall survival rates following exposure to high-concentration heavy metal solutions, with or without chelating treatments (Figure 5). These studies are critical to confirm the biocompatibility of the macromolecular conjugate and its effectiveness as a heavy metal chelator.

**Figure 5:**
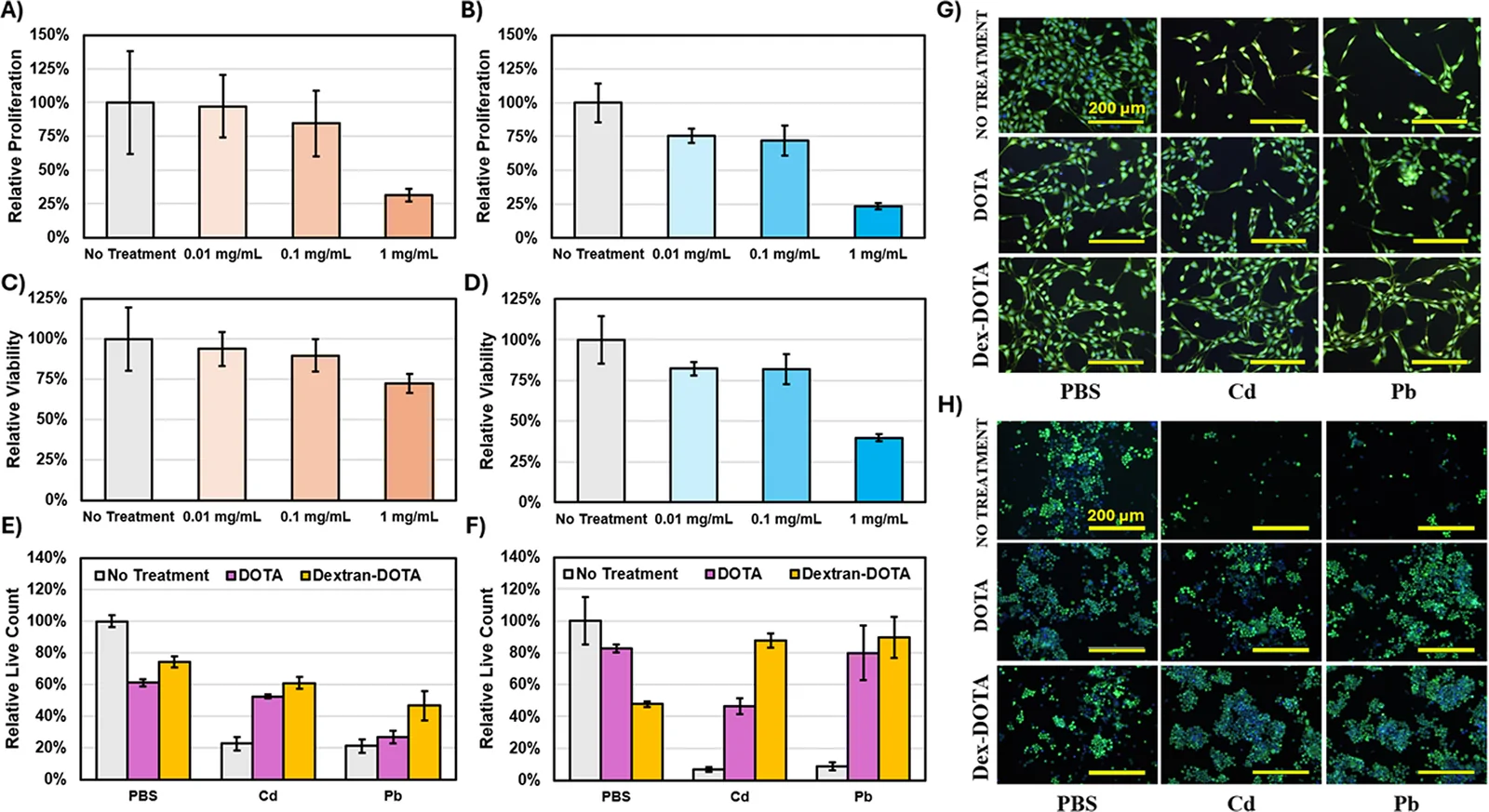
*In vitro* cytotoxicity of Dex-DOTA conjugated macromolecule at different concentrations of the chelator (DS ∼ 0.99; 1 mg/mL ∼ 1.8 mM). Proliferation (A, B), relative viability (C, D), and survival after metal toxin exposition (0.5 mg_Dex‑DOTA_/mL; 0.25 mg/mL of metal; E, F) for fibroblast (NIH/3T3) and macrophages (RAW 264.7) cells, respectively. Laser-scanning confocal microscopy images of cultured NIH/3T3 (G) and RAW 264.7 (H). Cells were labeled with calcein AM (green) for live cells and Hoechst (blue) for cell nuclei over 14 days.

Fibroblast proliferation (Figure 5A) revealed no significant deviations from the control at low-to-mid concentration systems, indicating that the Dex-DOTA conjugate does not inherently disrupt the proliferation of NIH/3T3 cells *in vitro*. However, at elevated concentrations, a significant attenuation of the cell proliferation was observed, resulting in a relative decrease of approximately 69.9% compared to the untreated control. Notably, cellular viability remained robust across the entire dose range (Figure 5C). This discrepancy between metabolic activity and membrane integrity suggests that the observed reduction in cell density at high concentrations is likely attributed to dose-dependent growth inhibition rather than acute cytotoxicity.

Evaluation of RAW 264.7 macrophage response revealed a dose-dependent effect on metabolic activity consistent with the fibroblast findings. At low-to-mid concentrations, macrophage proliferation (Figure 5B) exhibited no significant variation compared to the untreated control, indicating that the Dex-DOTA conjugate does not inherently perturb macrophage homeostasis *in vitro*. However, exposure to high concentrations of the macromolecular chelator elicited a significant reduction in proliferation capacity, with metabolic activity decreasing to 23.5% relative to the control. In contrast to the fibroblast results, this attenuation was accompanied by a significant decline in relative cell viability at high dosage (Figure 5D). These results suggest that elevated concentrations of the Dex-DOTA macromolecule may induce acute cytotoxicity in macrophage populations, potentially compromising the integrity of the immune microenvironment during high-dose chelating treatments.

Overall, while elevated concentrations of the Dex-DOTA conjugate appear to induce cytostatic effects on fibroblasts and macrophages, the macromolecular conjugate demonstrates an excellent safety profile within low-to-mid dosage range. The absence of discernible detrimental effects at these concentrations indicates a broad therapeutic window for the Dex-DOTA macromolecular chelator. These results suggest that Dex-DOTA is a viable candidate for prophylactic interventions or sustained chelation treatments, where moderate dosing can be utilized to ensure effective metal sequestration while maintaining systemic biocompatibility and preserving the integrity of the immune microenvironment.

Following the primary biocompatibility assessments, both NIH/3T3 fibroblast and RAW 264.7 macrophage lineages were subjected to heavy metal solutions to evaluate the protective efficacy of the Dex-DOTA chelator. Overall, a significant reduction in viable cell density relative to untreated control was observed across both cell lines upon exposure to the heavy metal solutions, confirming the strong cytotoxic effects of these metal toxins at high concentrations. These findings establish a baseline of metal-induced cellular damage, providing a robust model for assessing the subsequent rescue potential of the Dex-DOTA macromolecular chelator.

In the fibroblast model (Figure 5E,G), while monomeric DOTA exhibited a modest recovery of live cells compared to the untreated PBS control, the Dex-DOTA conjugate demonstrated a significant recovery in cell survival, comparable to that observed in the control. Furthermore, the Dex-DOTA treatment induces a statistically significant enhancement in cell survival following exposure to high concentrations of cadmium and lead. This superior cytoprotective effect suggests that the Dex-DOTA chelator is capable of rescuing fibroblast survival and proliferation, potentially by reducing heavy-metal induced oxidative stress and subsequent membrane damage as a consequence of Dex-DOTA metal complexation. Further studies are required to elucidate the precise intracellular mechanism driving the Dex-DOTA cytoprotective activity.

In the macrophage survival assays (Figure 5F,H), a disparity in therapeutic performance was observed under cadmium-induced toxicity. Monomeric DOTA failed to significantly increase the relative live count, a result that correlates with the limited cadmium sequestration presented by the unreacted macrocyclic chelator. In contrast, intervention with the Dex-DOTA chelator effectively reversed cadmium-induced cytotoxicity, restoring macrophage survival to levels comparable to PBS control. These results demonstrate the superior cytoprotective capacity of the macromolecular conjugate, particularly in neutralizing highly toxic heavy metals that are not effectively captured by small-molecule chelators.

Taken together, these results indicate that the Dex-DOTA macromolecule functions as a high-performance systemic chelating agent, with the potential to neutralize the damaging effects of cadmium and lead, offering a promising therapeutic strategy for the management of heavy metal poisoning. Consequently, Dex-DOTA emerges as a viable candidate for prophylactic interventions or sustained chelation treatments, where moderate dosing can be utilized to ensure effective metal sequestration while maintaining systemic biocompatibility.

### 3.6 *In Vivo* Compatibility Assays Using *Caenorhabditis elegans*

The comparison of the sequenced nematode and human genomes has established that *Caenorhabditis elegans* (*C. elegans*) possesses significant genetic homology to mammals.^50^ Beyond genomic similarities, *C. elegans* shares conserved cellular metabolic pathways and signaling mechanisms with humans, making the nematode a robust toxicological model for predicting systemic responses to chronic toxin exposure.^51,52^ The simple and compact platform of the organism facilitates rapid high-throughput screening in pharmacological studies.^53^ Notably, the high degree of conservation in neuronal and gastrointestinal functions renders *C. elegans* particularly indicative of neurotoxicity and oral administration outcomes.^54–56^

Consequently, *C. elegans* serves as an appropriate *in vivo* model to evaluate the efficacy of Dex-DOTA in mitigating heavy metal-induced behavioral and developmental toxicity (Figure 6). Utilizing this nematode framework allowed us to establish a robust, high-throughput *in vivo* proof-of-concept paired with our *in vitro* assays. Nevertheless, further testing in larger animal models is required to fully assess systemic efficacy, pharmacokinetics, and safety profiles, essential for future clinical translation.

**Figure 6:**
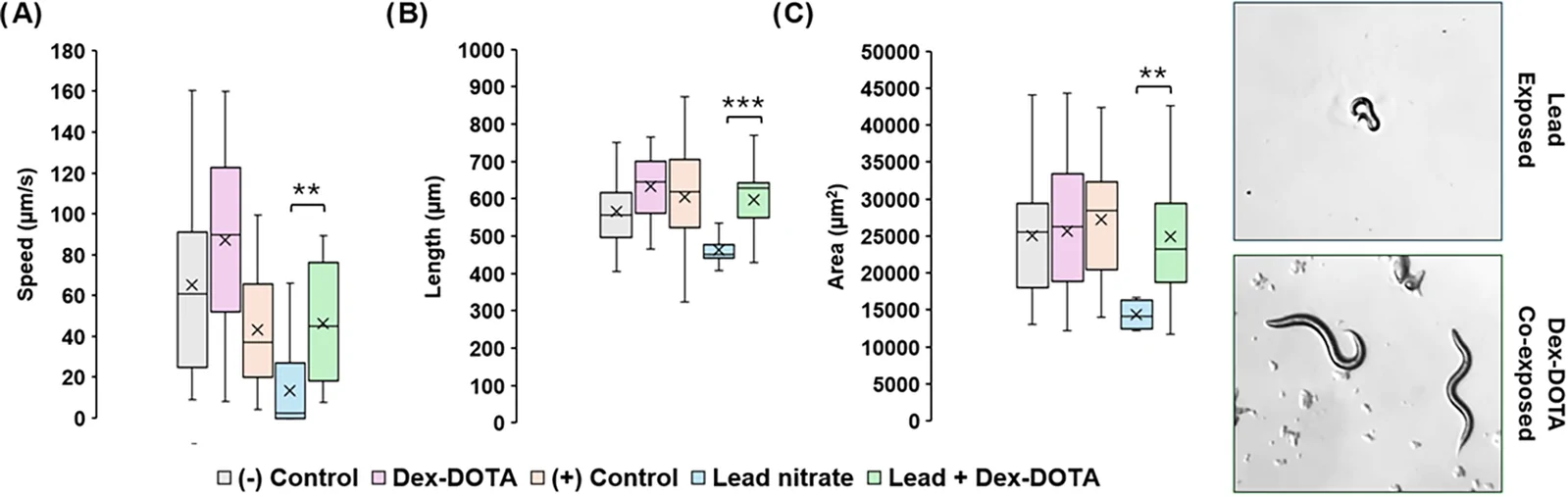
Dex-DOTA rescues lead-induced locomotor and growth deficits in C. elegans. (A) Speed (μm s^–1^), (B) body length (μm), and (C) body area (μm^2^) following 24 h acute liquid exposure to negative control (0.85% NaCl + OP50), Dex-DOTA (70 μM; DS ∼ 0.99), osmotic stress control (200 mM NaCl), lead nitrate (0.70 mM), or lead nitrate (0.70 mM) + Dex-DOTA (70 μM; DS ∼ 0.99; 1:10 molar ratio). Lead significantly reduced all three end points relative to the negative control. Co-exposure with Dex-DOTA significantly improved speed, length, and area relative to lead alone and was not different from the negative control. Data are presented as box-and-whisker plots (median, IQR, range); mean shown as (×). Statistical analysis was performed using Kruskal–Wallis with Dunn’s multiple comparisons. **p < 0.01; ***p < 0.001.

Across behavioral and morphological end points, statistically significant differences were observed between the lead only condition and the combined lead and Dex-DOTA treatment condition for speed (*p* = 0.0018), body length (*p* < 0.001), and body area (*p* = 0.0011). These comparisons suggest that Dex-DOTA treatment could protect organisms from the toxic effects of lead exposure.

Locomotor speed in *C. elegans* is a well-established quantitative measure of neuromuscular function and neuronal integrity.^57,58^ Neurotoxicity and environmental toxicology studies routinely use locomotion to assess chemical induced impairment of motor neuron signaling and muscle performance.^59^ Reductions in speed are consistently interpreted as evidence of neurotoxic or neuromuscular stress.^60^

In the present study, the lead only condition was statistically different from the negative control, demonstrating that 0.70 mM lead nitrate induced significant locomotor impairment under these exposure conditions, consistent with reports in literature of *C. elegans* locomotor impairment following exposure to lead nitrate.^61,62^

This 10-fold excess relative to the Dex-DOTA concentration was deliberately chosen to serve as an acute challenge dose. By overloading the system with an excess of toxic metal ions relative to the therapeutic chelating sites, we were able to rigorously evaluate the protective boundaries and remedial efficacy of the Dex-DOTA conjugate under severe, saturated metal-stress conditions. Additionally, compared to *C. elegans* environmental toxicology literature, exposure levels typically fall between 0.05 mM and 0.2 mM for standard assays designed to induce measurable acute neurobehavioral defects or severe locomotor toxicity.^59^

In contrast, the combined lead and Dex-DOTA treatment condition was not statistically different from the negative control, indicating protection of baseline movement behavior. The significant improvement relative to the lead only condition supports the conclusion that Dex-DOTA reduced the functional neurobehavioral consequences of lead exposure.

Body length and projected body area are established morphological end points in *C. elegans* developmental and toxicological research.^56^ Growth in this organism is tightly regulated by conserved genetic and metabolic pathways,^63,64^ and toxicant exposure frequently manifests as reduced body size due to impaired feeding, altered metabolism, or developmental delay.^65–67^ In this assay, worms exposed to lead alone exhibited statistically significant differences in both length and area relative to the negative control, indicating disruption of normal growth over the 24 h exposure period. This is consistent with literature reports of *C. elegans* morphology following exposure to lead nitrate.^68,69^ In contrast, the combined lead and Dex-DOTA treatment condition was not statistically different from the negative control for either morphological metric. The significant differences between the lead only and combined treatment groups confirm that Dex-DOTA attenuated lead-induced growth inhibition.

Importantly, the protective effect was observed under exposure conditions specifically designed to preserve lead bioavailability, including the use of phosphate-free buffer and standardized bacterial content. This minimizes the likelihood that the observed protection is attributable to precipitation artifacts or uncontrolled environmental sequestration. Rather, the data is consistent with chelation-mediated reduction of biologically available lead.

Collectively, these findings demonstrate that acute exposure to 0.70 mM lead nitrate produces statistically significant locomotor and morphological toxicity in synchronized young adult *C. elegans*, and that coadministration of Dex-DOTA significantly mitigates these effects. The absence of statistical differences between the combined lead and Dex-DOTA treatment condition and the negative control across speed, length, and area indicates effective functional and developmental protection under the defined experimental conditions.

## 4 Conclusions

In this study, a water-soluble macromolecule was successfully synthesized through the macromolecular conjugation of DOTA and dextran, providing a safer alternative for systemic chelation therapy.

The CDI-mediated coupling of DOTA to the polysaccharide backbone achieved high conjugation efficiency when utilizing a molar excess of both the macrocyclic chelator and the activating reagent, in conjunction with a prolonged activation of the carboxylic acid moieties. This observed trend underscores the tunability of the conjugation process, which allows for the precise tuning of the Dex-DOTA macromolecule to meet specific therapeutic requirements.

Furthermore, the Dex-DOTA macromolecular conjugate is stable across a range of biomimetic environments. This high level of stability is a crucial parameter for the development of orally administered systemic chelating agents, as it ensures the chemical and structural integrity of the conjugate throughout gastrointestinal and systemic transit.

The Dex-DOTA macromolecule demonstrates a marked affinity for heavy metals and improves the chelation performance over monomeric DOTA. While this preferential coordination of large metal ions is primarily governed by the DOTA macrocycle, its conjugation to the dextran backbone further optimizes the local coordination environment. This modification not only enhances the intrinsic affinity of DOTA for heavy metals but also effectively increases the overall chelation capacity of the macrocyclic chelator.

Our results suggest that Dex-DOTA exhibits an excellent safety profile within the therapeutic range. The macromolecular conjugate was found to be cyto-compatible within the low-to-mid dosage range *in vitro*, demonstrating superior cytoprotective properties over monomeric DOTA. Similar protective activity was observed *in vivo*, where the presence of Dex-DOTA mitigates the toxic effects in *C. elegans* models induced by heavy metal exposure. Taking together, these results suggest future *in vivo* studies with larger models will demonstrate good compatibility.

The robust protective action against heavy metal-induced damage, and strong biocompatibility, suggests a broad therapeutic window for the Dex-DOTA macromolecular chelator. By integrating high-affinity coordination chemistry with a biocompatible polysaccharide template, this Dex-DOTA conjugate represents a significant advancement in the design of systemic chelation agents. This approach provides a promising therapeutic strategy for the management of heavy metal poisoning, offering a safer alternative for both prophylactic and sustained chelation treatments.

## Supplementary Material



## Notes

*C. elegans* data analysis and experimental instrumentation, calibration curves for UV–vis analysis, Doble-reciprocal analysis for the chelating saturation curves and the obtained parameters, and the volumetric fraction of Dex-DOTA conjugates in function of the particle size after incubation in different SBF (PDF)
