# Supplementary material for: Heavy Metal Chelation and Bioprotective Activity of Dextran-DOTA Macromolecular Conjugate
Source: Biomacromolecules. 2026 Aug 5;27(9):6009–22. doi: 10.1021/acs.biomac.6c00847 (PMC13580026; doi:10.1021/acs.biomac.6c00847)
Supplement: Supplementary file 1 [file bm6c00847_si_001.pdf]

Supporting Information

Heavy Metal Chelation and Bioprotective Activity of Dextran-DOTA Macromolecular Conjugate

Sebastian Guajardo[a], Hunter Wood[a], Chenyun Deng[a], Neo Alpha[a], Stefanie A. Sydlik [a,b,#]

[a]Department of Chemistry, Carnegie Mellon University, Pittsburgh, PA, USA

[b]Department of Biomedical Engineering, Carnegie Mellon University, Pittsburgh, PA, USA

Highlights:

- Covalent conjugation of DOTA onto a Dextran backbone creates a stable macromolecular chelator.
- Dex-DOTA exhibits superior heavy metal affinity and sequestration compared to monomeric DOTA.
- Dex-DOTA elicits robust colloidal stability across diverse physiological conditions.
- Dextran-DOTA demonstrates high cytocompatibility and bioprotection against heavy metal toxicity.
- Refined sequestration provides potent bioprotection, reducing heavy metal stress in *C. elegans*.

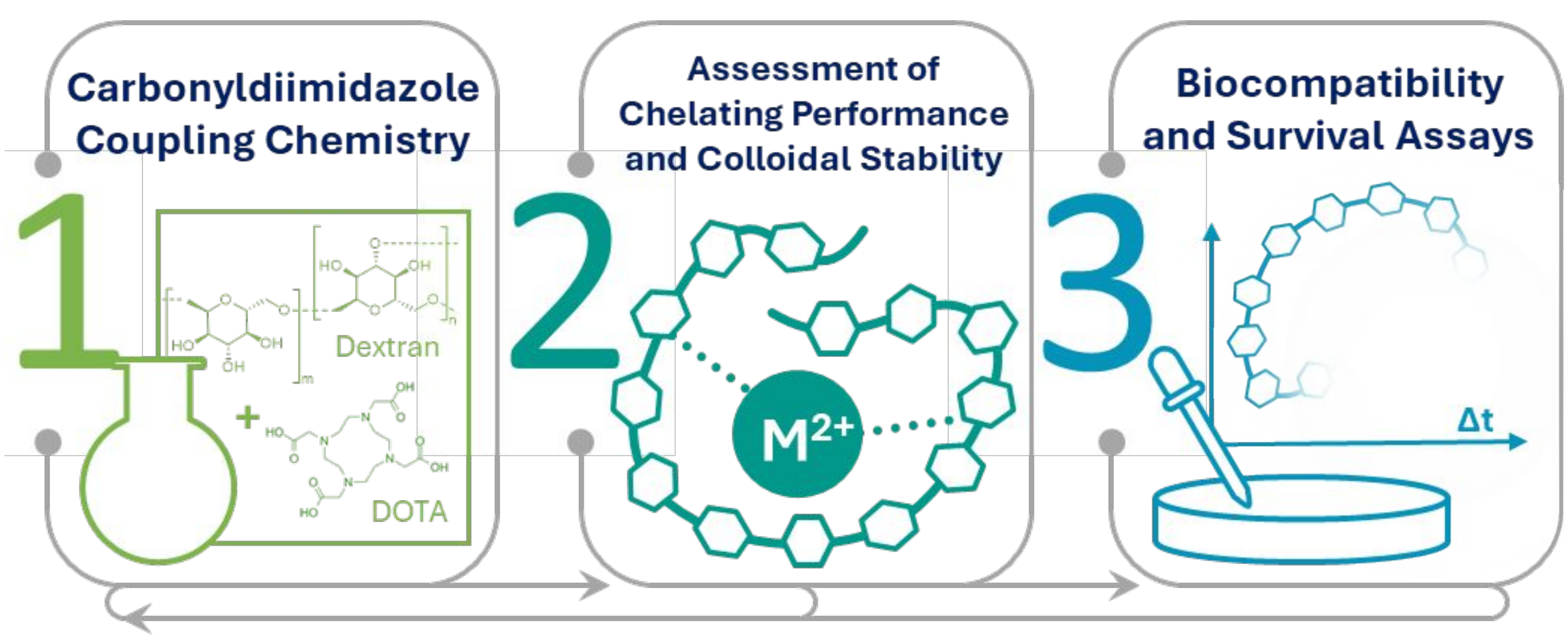

Table of Contents

Experimental section (Complementary):

Synthesis of Dex-DOTA conjugate: In a typical synthesis, 972.9 mg of 1,1-carbonyldiimidazole (CDI, 6.0 mmol) is dissolved in 50 mL of DMSO at 80°C. Once dissolved and at the desired temperature, 404.4 mg of 1,4,7,10-tetraazacyclododecane-1,4,7,10-tetraacetic acid (DOTA, 1.0 mmol) DOTA is further added for activation and let under constant stirring for 3 hours or until the solution turns brown, without any sign of undissolved DOTA. Following the activation step, 81.1 mg of dextran (0.5 mmol; anhydro glucose unit molecular weight: 162.14 mg $mmol^{-1}$) is added along with a catalytic amount of 1-hydroxybenzotriazole (HOBt, 13.5 mg, 0.1 mmol). The reaction proceeds over 18 hours at 80 °C under constant stirring with a condenser to avoid loss of volume. Following the reaction, the product is poured into 300 mL of methanol, letting the product precipitate. Once the supernatant is carefully discarded, the collected precipitate undergoes repeated cycles of ethanol washing and centrifugation. After the product is thoroughly washed, the precipitate is dissolved in 30 mL of DI water and dialyzed against pure DI water over five days, changing the DI water daily. Finally, the purified product is frozen and lyophilized. The dried samples were prepared for further chemical characterization (NMR, FTIR, XPS, and SEM-EDX).

Table 1: Synthesis conditions and degree of substitution of Dex-DOTA

DOTA (mol)	CDI (mol)	Dextran (mol_{AGU})	$Time_{Act.}$ (h)	N:C	DS (mol_{DOTA}/mol_{AGU})
1	4	1	0.25	0.07	0.15 ± 0.03
1	6	1	0.25	0.10	0.26 ± 0.04
1	6	0.5	1.00	0.16	0.66 ± 0.13
1	6	0.5	3.00	0.18	0.99 ± 0.18

$$DS = \frac{n_{C,Dextran} \cdot \%N}{n_{N,DOTA} \cdot \%C - n_{C,DOTA} \cdot \%N} \quad ; \quad \left[\frac{mol_{DOTA}}{mol_{AGU}}\right]$$

$n_{C,Dextran}$: number of carbon atoms in a dextran unit
$n_{N,DOTA}$: number of nitrogen atoms in a DOTA molecule
$n_{C,DOTA}$: number of carbon atoms in a DOTA molecule
%C, %N: atomic percentage of carbon or nitrogen in the sample

$Time_{Act.}$: Time of activation; mol_{AGU}: moles of anhydrous glucose unit.

Analysis of C. elegans behavior and morphology: Worms from each treatment well used for exposure testing were analyzed to determine whether Dex-DOTA affected *C. elegans* behavior or morphology. To acquire videos, each equilibration plate was analyzed separately. 8-10 worms were transferred using a platinum wire to a fresh NGM plate without an OP50 *E. coli* bacterial lawn. The plate was marked with a 5 mm line to ensure appropriate video scaling. Next, three 30-second videos (30 fps) were recorded using an iPhone 13. This gave nine 30-second videos per treatment condition (3 wells per treatment condition x 3 videos per treatment condition). The videos were converted from .mov to .avi for compatibility with ImageJ. Using ImageJ, the scale of each video was determined in pixels/mm according to the scale bar which was visible in each

video. Having determined the scale of each video, behavioral and morphological features could be extracted from the videos using a free worm tracking software called TierpsyTracker which was developed by Imperial College in London.[1] The software GUI includes four options upon startup. The first option is "set parameters" in which the minimum area, maximum area, block size, threshold, dilation, frames per second, and scale (micron/pixel) were manually optimized based on each video to ensure proper worm tracking. The second option is "batch processing multiple files" in which the video is analyzed by the software according to the settings stipulated in the "set parameters" file. The third option is "tierpsy tracker viewer" which allows the user to visualize the software tracking the worms in real time. At this step, if the software did not properly track worms, settings in the parameters file were further changed to ensure accurate tracking. The last option is "get features summary" in which the raw data is exported to a .csv file. *C. elegans* videos were analyzed according to this workflow, which returned a variety of quantitative behavioral and morphological data including speed, fraction of forward movement, length, curvature, and area. Interquartile range was used to determine the presence of outliers in each data set. Data for each treatment condition was pooled and plotted together. Following outlier identification, statistical significance was determined using the Kruskal-Wallis nonparametric test followed by Dunn's post hoc analysis.

Instrumentation: X-ray photoelectron spectroscopy (XPS): XPS data for the Dex-DOTA samples was acquired on an ULVAC-PHI Genesis system with an Al k-α source, X-ray power 100μm 25W 15kV, at a 280-eV pass energy, 20 ms dwell time, and 0.1 eV increment. Deconvolution was performed using the tool *Peak analyzer* in the software *OriginPro* under student license. In case of significant shift of the overall C1s peak, from the standard 284.8 eV, the data were corrected previous deconvolution, so the maximum point match the standard. *Fourier-transform infrared spectroscopy (FTIR) spectroscopy:* A PerkinElmer Frontier FTIR Spectrometer with an attenuated total reflectance attachment containing a germanium crystal was used to perform FTIR spectroscopy. Raw spectra were obtained over a range of 4000–700 cm^{-1} with 4 cm^{-1} resolution and analyzed on Spectrum software (PerkinElmer). Each spectrum was ATR corrected followed by a baseline correction. *Scanning Electron Microscopy coupled with Energy Dispersive X-ray spectroscopy (SEM-EDX):* SEM images were acquired using a FEI Quanta 600 FE SEM equipped with a SE Everhart Thornley Detector for secondary electron (SE) images and a Scintillator Backscatter Detector for backscatter electron (BSE) images. EDX data was acquired with an Oxford INCA EDS System full analytical XMAX 80mm SDD EDX detector. *Nuclear Magnetic Resonance (NMR)*: A 500 MHz NEO Bruker NMR spectrometer and *TopSpin* software were used to acquire NMR spectra of reactants and products. Deuterium oxide was used as solvent. *TopSpin* was used to extract data from NMR experiments and further plotted using *OriginPro* under student license. *Dynamic Light Scattering (DLS):* DLS data for colloidal stability analysis was taken on a Malvern Panalytical Zetasizer Ultra taking a 60 second temperature equilibration time at 25 °C prior to each run which consisted of 5 scans with 1.49 as refractive index. Different simulated body fluids (PBS, SGF, and SIF) were used as solution media. Samples were incubated at 37 °C for different time periods prior to DLS scans.

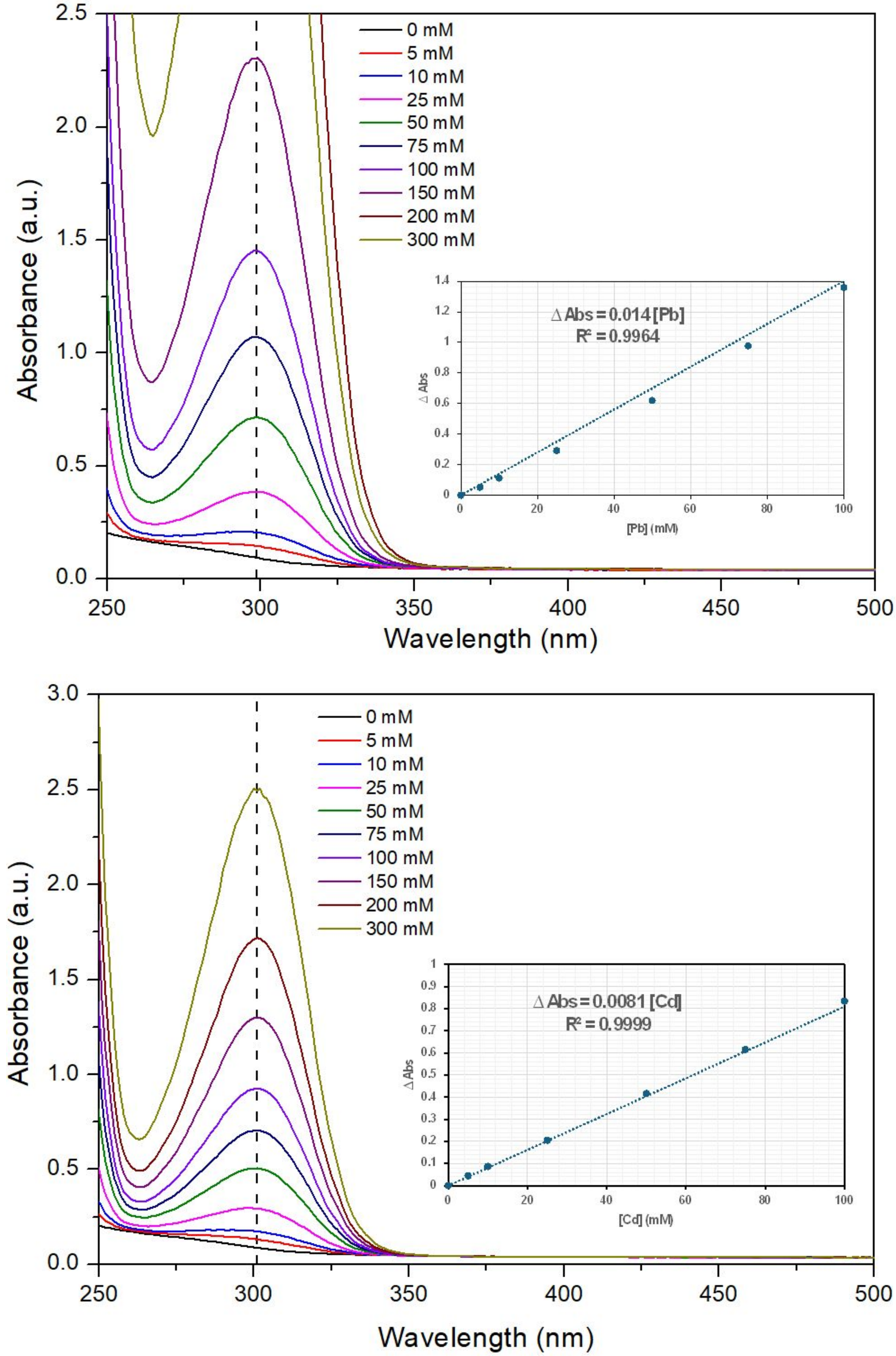

Figure S1. UV-Vis calibration curves for lead (Upper) and cadmium (Bottom).

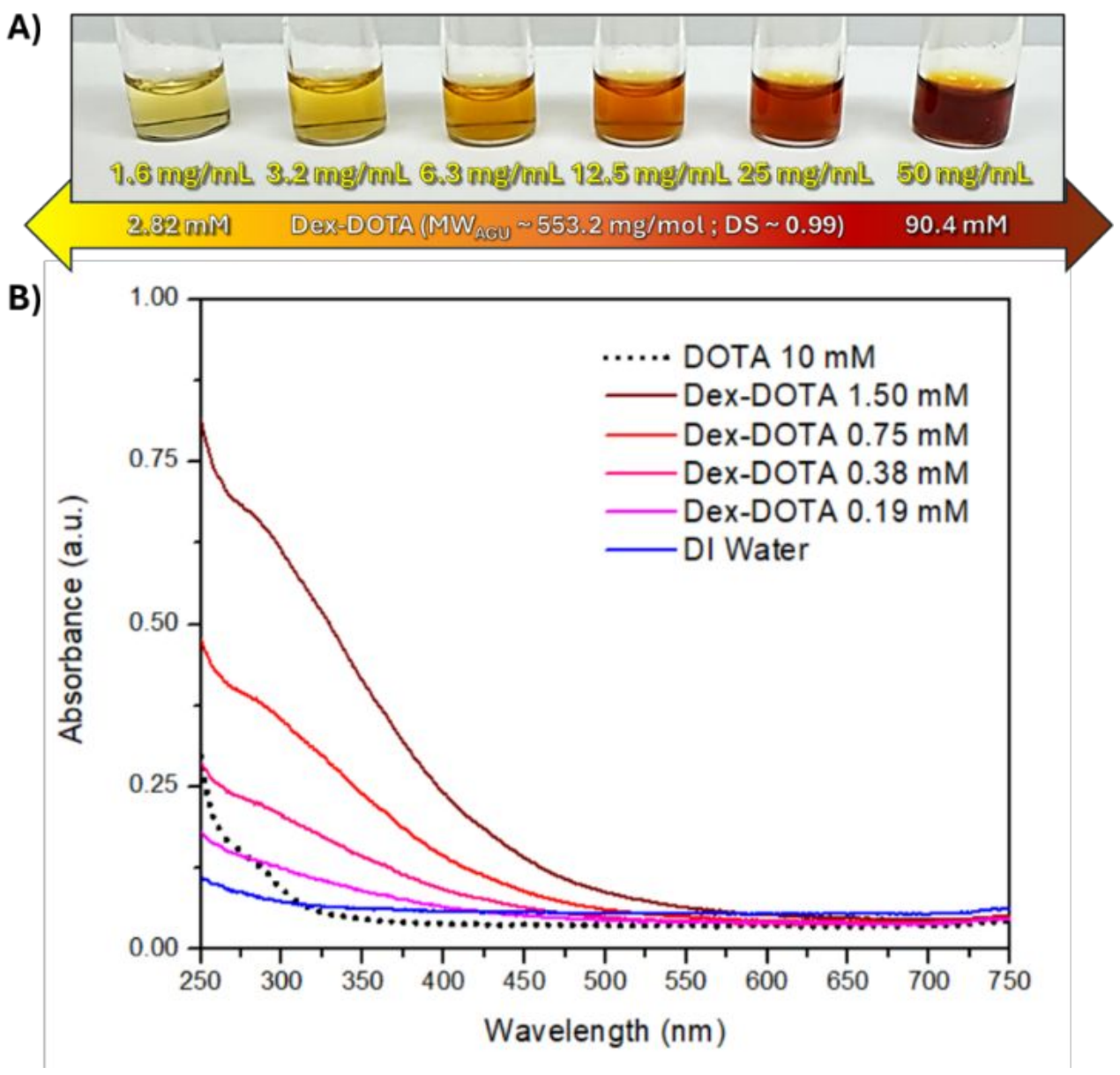

Figure S2. Dex-DOTA solubility and absorbance references. Dilution of Dex-DOTA (DS ~ 0.99), solutions up to 50 mg/mL (~90.4 mM) (A), and UV-visible spectra for Dex-DOTA (DS ~ 0.99) at different concentrations and DOTA solution as references.

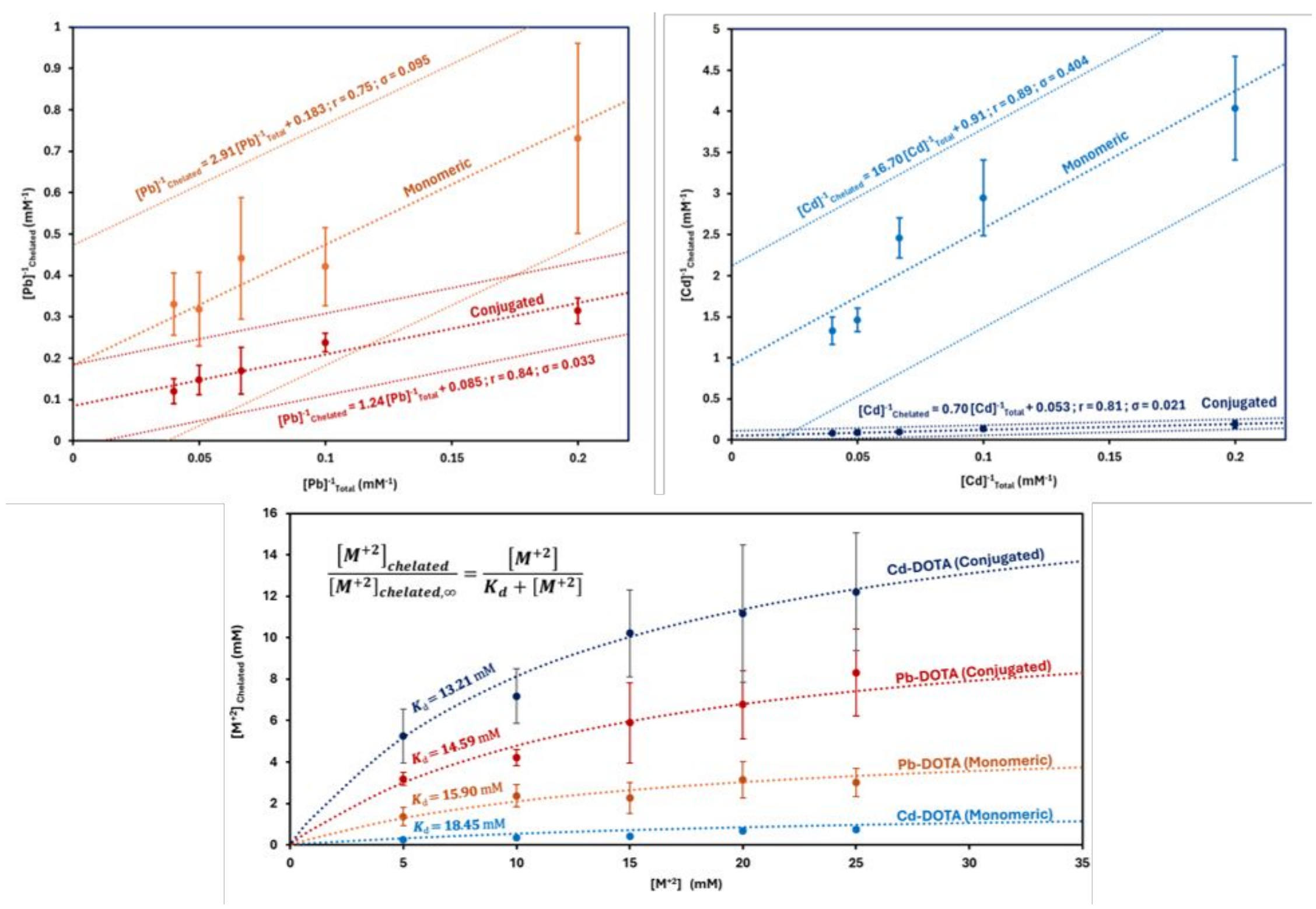

Figure S3. Double-reciprocal analysis of the chelating saturation curves and affinity comparisons for lead (left) and cadmium (right) using free DOTA and the Dex-DOTA conjugate (1.25 mM, DS ~ 0.99). Initial estimates for maximum capacity and K_d were extracted via double-reciprocal linearization and subsequently optimized by minimizing the sum of squared residuals for the Hill-Langmuir equation. Dotted lines represent the 95% confidence intervals of the linear regression (p-value < 0.05).

Table S2: Parameters for binding saturation model (Double-reciprocal analysis)

Chelator	Heavy Metal	K_d (mM)	Saturation** (mM)
DOTA	Lead	15.90	5.46
	Cadmium	18.45	1.10
Dex-DOTA*	Lead	14.59	11.76
	Cadmium	13.21	18.87

*Dex-DOTA with DS ~ 0.99 for direct comparison; **Predicted saturation for 1.25 mM of chelator.

Table S2: Parameters for binding saturation model (Hill-Langmuir equation)

Chelator	Heavy Metal	K_d (mM)	Saturation** (mM)
DOTA	Lead	16.14	5.38
	Cadmium	20.10	1.23
Dex-DOTA*	Lead	14.84	11.76
	Cadmium	10.18	17.08

*Dex-DOTA with DS ~ 0.99 for direct comparison; **Predicted saturation for 1.25 mM of chelator.

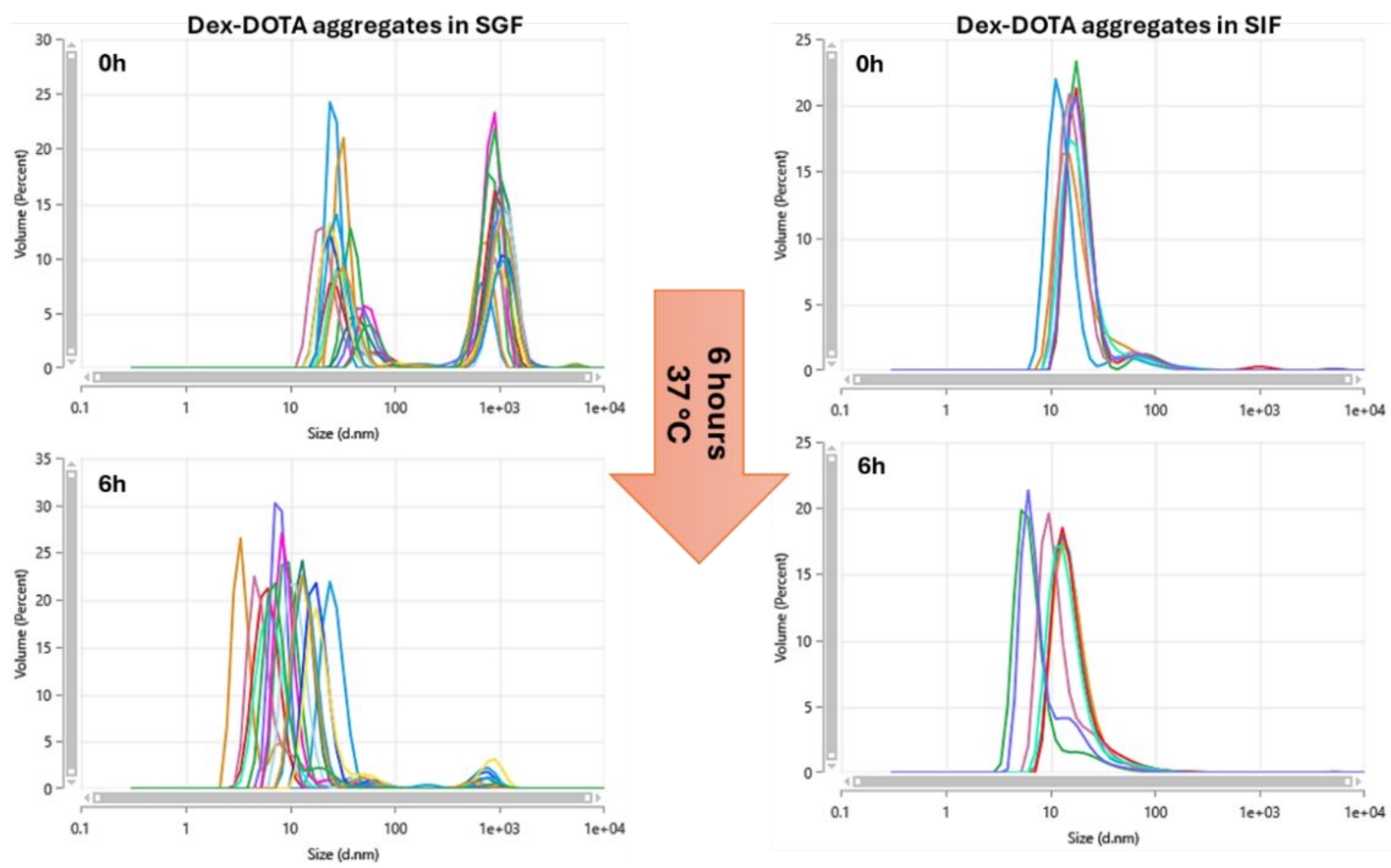

Figure S4. Volumetric fraction Dex-DOTA conjugates in function of the particle size after incubation in simulated gastric fluid (SGF, pH ~ 1.2, left) and simulated intestinal fluid (SIF, pH ~ 6.8, right) at 37 °C for 6 hours. Variation in colors represents the analysis for different replicates.

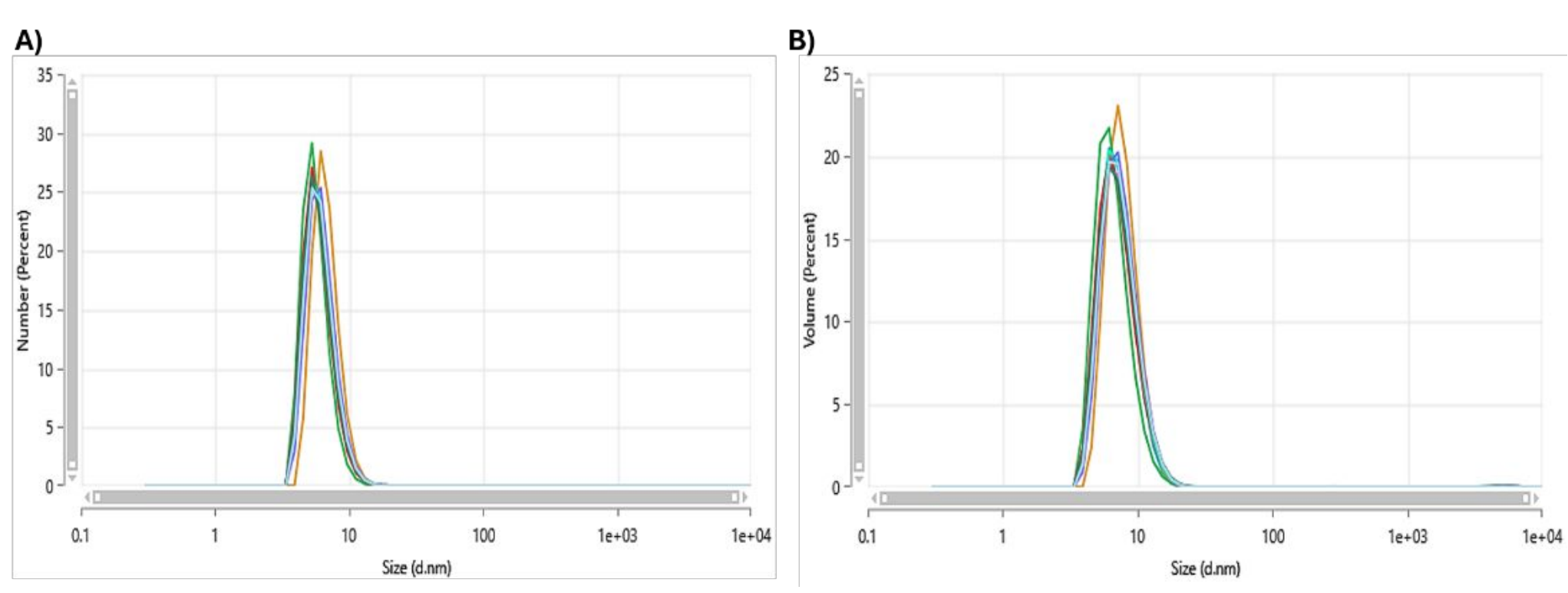

Figure S5. Numeric (A) and Volumetric fraction (B) of dextran in function of the particle size. The polysaccharide averages a particle size of 6.14 ± 0.31 nm. Variation in colors represents the analysis for different replicates.

References:

(1) Javer, A.; Currie, M.; Lee, C. W.; Hokanson, J.; Li, K.; Martineau, C. N.; Yemini, E.; Grundy, L. J.; Li, C.; Ch'ng, Q.; Schafer, W. R.; Nollen, E. A.; Kerr, R.; Brown, A. E. An Open Source Platform for Analyzing and Sharing Worm Behavior Data. *Nat Methods* **2018**, *15* (9), 645–646. https://doi.org/10.1038/s41592-018-0112-1.
